# Redox buffering and H_2_O_2_ orchestrate the vegetative development of *Marchantia polymorpha*

**DOI:** 10.1111/tpj.70317

**Published:** 2025-07-18

**Authors:** Cilian Kock, Judith Helmig, Nora Gutsche, Tom Dierschke, Stefanie J. Müller‐Schüssele, Sabine Zachgo

**Affiliations:** ^1^ Division of Botany Osnabrück University Osnabrück Germany; ^2^ Institute of Plant Biology and Zürich‐Basel Plant Science Centre University of Zürich Zürich Switzerland; ^3^ Molecular Botany, Department of Biology RPTU Kaiserslautern‐Landau Kaiserslautern Germany

**Keywords:** *Marchantia polymorpha*, meristem, roGFP2, HyPer7, redox, development, glutathione, ROS

## Abstract

Redox processes and reactive oxygen species (ROS) signaling play not only a crucial role in stress responses but also in angiosperm development. However, the specific mechanisms by which redox homeostasis regulates meristems and growth in non‐vascular plants remain poorly understood. Here, we demonstrate the applicability of the roGFP2‐hGrx1 and HyPer7 redox‐biosensors for imaging dynamic glutathione (GSH) and H_2_O_2_ redox states in the liverwort *Marchantia polymorpha*. RoGFP2‐hGrx1 microscopy, together with analysis of knockdown plants of the *GAMMA GLUTAMYLCYSTEINE SYNTHETASE* gene Mp*GSH1*, unveiled a more reduced GSH redox potential (*E*_GSH_) in the meristematic region and a more oxidized state in differentiated thallus tissues. Rather than absolute *E*_GSH_ values, maintenance of a GSH redox gradient is crucial for proper vegetative development. High‐resolution HyPer7 analysis detected a heterogenous H_2_O_2_ accumulation. Overall, the meristematic region exhibits lower H_2_O_2_ levels. Notably, a small zone with higher sensor oxidation is localized in the center of the meristem, likely comprising stem cells and proliferating derivatives. In differentiated thallus tissue, higher levels of H_2_O_2_ were detected. External H_2_O_2_ application revealed dose‐dependent effects that promote or arrest growth. Overproliferation in the meristematic region, driven by treatment with the CLAVATA3/EMBRYO SURROUNDING REGION‐related peptide (MpCLE2p) increased H_2_O_2_ levels in expanded meristems, supporting the importance of H_2_O_2_ signaling in balancing cell proliferation and differentiation in *M. polymorpha*. Further comparative high‐resolution redox sensor studies in bryophytes and vascular plants can shed light on the contribution of redox processes to the regulation of developmental processes and the formation of increasingly complex land plants.

## Significance Statement

Advanced redox sensors were established for in vivo monitoring of the glutathione redox buffer and the ROS H_2_O_2_ in the liverwort *Marchantia polymorpha*. High‐resolution microscopy data highlight the importance of dynamic redox signaling in the control of cell proliferation and differentiation processes. Formation of a glutathione redox gradient and accumulation of H_2_O_2_ in the proliferating stem cell zone affect meristems and thereby growth in this liverwort.

## INTRODUCTION

The evolution of land plants marks a pivotal transition in Earth's history where plants adapted to novel, challenging environments. Terrestrialization involved significant morphological and physiological plant innovations, including more intricate meristems that contributed to the formation of increasingly complex plants (Bowman et al., 2017; de Vries & Archibald, 2018; Harris et al., 2022). Reactive oxygen species (ROS), which are classically known as signaling molecules in stress responses, are receiving increasing attention due to their regulatory functions in developmental processes (Ali & Muday, 2024; Becker et al., 2025; Mittler, 2017; Peláez‐Vico et al., 2024; Zhou & Dresselhaus, 2023). Land plants realize their indeterminate growth by generative centers, meristems, that comprise stem cells and their daughter cells that give rise to all other plant tissues. Understanding the roles of redox buffers and ROS in stress and developmental processes in bryophytes sheds light on how these organisms have overcome the challenges of a terrestrial lifestyle and evolved novel plant structures that contributed to the diversity of modern ecosystems.

ROS, including hydrogen peroxide (H_2_O_2_), superoxide (O_2_^•−^) and hydroxyl radicals, are generated during aerobic metabolism in chloroplasts, mitochondria, and peroxisomes, or alternatively in the apoplast, and regulate metabolic processes and participate in stress responses (Mittler et al., 2022). However, accumulating evidence from angiosperms demonstrates that ROS also function as signaling molecules regulating meristem activities (Tsukagoshi et al., 2010; Zeng et al., 2017). In particular, H_2_O_2_ has garnered significant interest due to its unique properties, including relatively low reactivity and enhanced stability compared to other ROS (Noctor et al., 2018). Land plant meristems form stem cells, which maintain stemness and generate daughter cells that further divide and differentiate, thereby forming tissues and complex organs. Angiosperms, such as *Arabidopsis thaliana*, possess two primary types of meristems: the shoot apical meristem (SAM) and the root apical meristem (RAM). The SAM, located at the shoot apex, generates all above‐ground organs, comprising a central zone of stem cells surrounded by a peripheral zone where organ primordia form. In the RAM, the quiescent center (QC) and adjacent cells establish the stem cell niche. In both meristems, localized O_2_^•−^ accumulation in the central zone or QC supports stem cell maintenance, while elevated H_2_O_2_ levels in the peripheral or transition zone promote differentiation (Considine & Foyer, 2021; Tsukagoshi et al., 2010; Zeng et al., 2017). A specific ROS accumulation within the SAM and RAM is essential for balancing stem cell self‐renewal and differentiation, and disruptions in ROS balance can lead to loss of stem cell identity and premature differentiation. The tripeptide glutathione (GSH) is the most abundant thiol in plant cells and an important antioxidant, functioning as an electron donor in numerous metabolic processes as well as sustaining cellular redox homeostasis (Noctor et al., 2024). Under oxidative stress conditions, GSH is converted to glutathione disulfide (GSSG), which can be reduced back to GSH by glutathione reductases, thereby maintaining cellular redox homeostasis and preventing protein thiol oxidation. Mutants with reduced GSH levels in *A. thaliana*, such as *root meristemless1* (*Atrml1*) (Vernoux et al., 2000) and *zinc tolerance induced by iron 1* (*Atzir1*) (Shanmugam et al., 2012), exhibit severe root growth defects, highlighting the pivotal role of GSH in meristem development. Maintenance of a GSH buffer system and an optimal ROS balance in angiosperms is thus crucial for effective signaling in plant stress and development, underscoring an intricate relationship between oxidative stress management and cell division and differentiation processes.

Several meristem regulators that belong to conserved gene families such as CLAVATA (CLV), WUSCHEL (WUS)‐RELATED HOMEOBOX (WOX), KNOTTED1‐LIKE HOMEOBOX, and LEAFY were extensively characterized in angiosperms in the past decades. Advances in the establishment of molecular tools for *Marchantia polymorpha* enabled functional gene analyses and provided insights into the diversification of regulatory land plant gene activities. So far, studies in this liverwort and the moss *Physcomitrium patens* mainly focused on known conserved regulator homologs, which provided interesting insight into regulatory commonalities and differences during the evolution of land plant meristems (Arnoux‐Courseaux & Coudert, 2024; Fouracre & Harrison, 2022; Hata & Kyozuka, 2021). However, knowledge about the crucial role of ROS and redox processes in meristem and growth regulation has thus far been largely restricted to analyses in angiosperms. In contrast to vascular plants, the dominant phase of the life cycle in bryophytes is the haploid gametophyte. The thalloid liverwort *M. polymorpha* produces clonal asexual propagules called gemmae, disk‐shaped structures with two dormant meristematic regions. After dispersal from the gemma cups, gemmae undergo a juvenile meristem phase with anatomical rearrangements, and within the first week, a mature meristem is formed that produces a flat and horizontally growing thallus. Differently, angiosperm meristems often exhibit a radial symmetry and produce upright stems and roots (Moubayidin & Ostergaard, 2015; Shimamura, 2016; Spencer et al., 2024). In the center of the thallus notch of *M. polymorpha*, there is at least one wedge‐shaped stem cell with four cutting faces. This cell, also referred to as the apical cell, produces actively dividing daughter cells known as merophytes, which together form the stem cell zone (SCZ) (Shimamura, 2016). Thereby, the meristem of the *M. polymorpha* thallus disperses centrifugally novel cells that start to differentiate, generating dorsal and ventral structures such as the air pores and scales. During the vegetative growth phase, the thallus expands through periodic dichotomous branching from bifurcating meristems (Arnoux‐Courseaux & Coudert, 2024; Kohchi et al., 2021).

In this study, we unveiled the in vivo dynamics of the GSH redox buffer and the ROS H_2_O_2_ in *M. polymorpha* with an unprecedented tissue resolution using the genetically encoded redox sensor roGFP2‐hGrx1 (Albrecht et al., 2014; Aller et al., 2013; Müller‐Schüssele et al., 2021) and HyPer7, an improved H_2_O_2_ biosensor (Pak et al., 2020). Additionally, Mp*GSH1* knockdown plants were generated to analyze the role of GSH during vegetative growth. Our findings reveal distinctive states of the GSH redox buffer and H_2_O_2_ in the meristematic region and differentiated tissue, exhibiting crucial roles for growth control in this liverwort. Comparison of redox buffer and ROS activities in evolutionary informative plant lineages enables understanding their function and contribution to the formation of more complex body plans and thus land plant diversification.

## RESULTS

### Establishment of the GSH redox biosensor roGFP2‐hGrx1 in *M. polymorpha*

GSH participates in the maintenance of cellular redox homeostasis, which is crucial for various plant functions, including *A. thaliana* meristem activities and plant growth processes (de Simone et al., 2017). Here, we aimed to analyze GSH redox potentials (*E*_GSH_) in cell proliferation and differentiation processes in the liverwort *M. polymorpha*. Transgenic *M. polymorpha* plants were generated expressing the redox‐sensitive fluorescent sensor roGFP2 linked to the human glutaredoxin1 (hGrx1). RoGFP2 is a modified GFP featuring two additional cysteine residues, which undergo conformational changes upon GSH/GRX‐dependent oxidation and reduction (Albrecht et al., 2014; Hanson et al., 2004; Meyer et al., 2007; Schwarzländer et al., 2008). These structural rearrangements affect its fluorescence properties and enable ratiometric analysis of the *E*_GSH_ (Meyer et al., 2007). RoGFP2 has been N‐ or C‐terminally fused to the human glutaredoxin1 (hGrx1), resulting in a highly specific and sensitive GSH biosensor shown to facilitate rapid equilibration between the sensor protein and the redox state of the cellular GSH pool (Aller et al., 2013; Gutscher et al., 2008). This overcomes kinetic limitations of the roGFP2/GRX interaction and constraints caused by the absence of appropriate GRX activities (Aller et al., 2013; Gutscher et al., 2008; Meyer & Dick, 2010). The roGFP2‐hGrx1 sensor was expressed under the control of the Mp*EF1α* promoter mediating constitutive expression (Althoff et al., 2014) and one male and one female transgenic line with strong ubiquitous roGFP2‐hGrx1 fluorescence were selected for further analyses.

**Figure 1:**
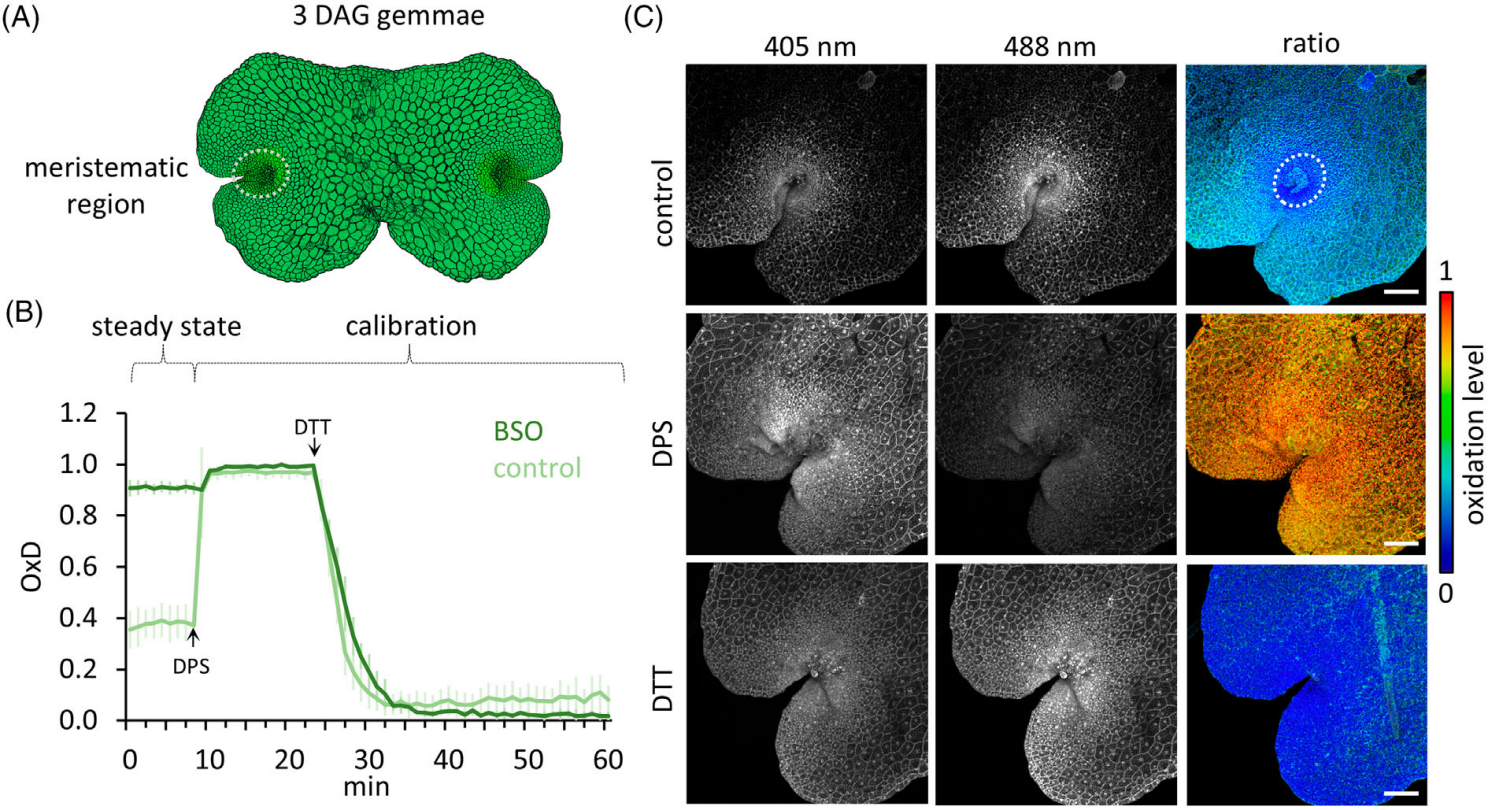
Analysis of the roGFP2‐hGrx1 redox sensor in *Marchantia polymorpha*. (A) Schematic representation of *M. polymorpha* gemmae 3 days after germination (DAG), indicating the meristematic region investigated by microscopic analyses. (B) Plate reader measurements of roGFP2‐hGrx1 fluorescence intensity in 3 DAG gemmae. Fluorescence emission was measured after 405/488 nm excitation, and the degree of oxidation (OxD) was calculated. Results are compared between control plants and plants treated with 500 μM glutathione (GSH) synthesis inhibitor L‐buthionine‐(S,R)‐sulfoximine (BSO). Calibration was performed using 2 mM 2,2′‐dipyridyldisulfide (DPS) for oxidation and 20 mM dithiothreitol (DTT) for reduction. Data were collected from one male and one female line (*n* = 9). (C) Microscopic images of roGFP2‐hGrx1 fluorescence in 3 DAG gemmae. Images show control gemmae (top panel), where the GSH redox sensor is more reduced in the meristematic region compared to differentiated thallus cells with a higher oxidized state. This spatial GSH buffer disparity was abrogated by 2 mM DPS and 20 mM DTT treatments, causing uniformly distributed high and low sensor oxidation levels, respectively. One male and one female line were investigated (*n* = 15). Calibration bar: rainbow false color representing roGFP2‐hGrx1 oxidation level, ranging from blue (fully reduced) to red (fully oxidized). All images are maximum intensity *Z*‐stack projections. Scale bars: 100 μm.

Firstly, roGFP2‐hGrx1 was validated as an eligible redox sensor in *M. polymorpha* by in vivo responsiveness to the thiol oxidant 2,2′‐dipyridyl disulfide (DPS) and the reductant dithiothreitol (DTT) using plate reader measurements to implement a high‐throughput approach for quantitative real‐time monitoring of redox changes (Wagner et al., 2019). Measurements were conducted with gemmae 3 days after germination (3 DAG) comprising two meristematic regions with high cell division activity, ultimately producing differentiated thallus cells (Figure 1A). In order to characterize in vivo the dynamic range of roGFP2‐hGrx1, the sensor was fully oxidized with DPS and thereafter reduced with DTT (Figure 1B). RoGFP2‐hGrx1 was predominantly reduced in 3 DAG control plants with a degree of oxidation (OxD) of 0.37, measured as described by Aller et al. (2013).

To elucidate the effects of a decreased GSH pool on the redox potential, the GSH synthesis inhibitor L‐buthionine‐(S,R)‐sulfoximine (BSO) was used, shown to deplete GSH in *A. thaliana* (Vernoux et al., 2000). After a 3‐day treatment with 500 μM BSO, the OxD increased up to 0.90 in WT gemmae, demonstrating the requirement of appropriate GSH levels to maintain a reducing *E*_GSH_ (Figure 1B). The plate reader measurements demonstrate the applicability of the roGFP2‐hGrx1 sensor in *M. polymorpha* gemmae and its suitability to investigate responses to altered redox conditions.

To enhance the spatial resolution and investigate the cellular GSH redox status in distinct areas of 3 DAG gemmae, we next conducted ratiometric roGFP2‐hGrx1 microscopy and detected fluorescence signals in the cytosol and nuclei (Figure S1a). Microscopic analyses revealed the lowest roGFP2‐hGrx1 oxidation level in the meristematic region with a high proliferation activity, comprising stem cells, merophytes, and their derivatives. Oxidation levels increased in the surrounding differentiated cells (Figure 1C). Calibration with DPS mediated a strong and uniform increase in oxidation throughout the whole gemmae, while DTT application resulted in an overall decreased roGFP2‐hGrx1 oxidation level (Figure 1C).

Taken together, the roGFP2‐hGrx1 sensor dynamically detects the *E*_GSH_ in *M. polymorpha* and enables in vivo recording of the intracellular redox state with a high tissue resolution. RoGFP2‐hGrx1 analyses revealed distinct oxidation differences between the meristematic region and tissue differentiation regions in the thallus, suggesting a participation of redox regulation in these processes in *M. polymorpha*.

### Mp*GSH1* knockdown plants reveal a crucial GSH buffer function in *M. polymorpha*

**Figure 2:**
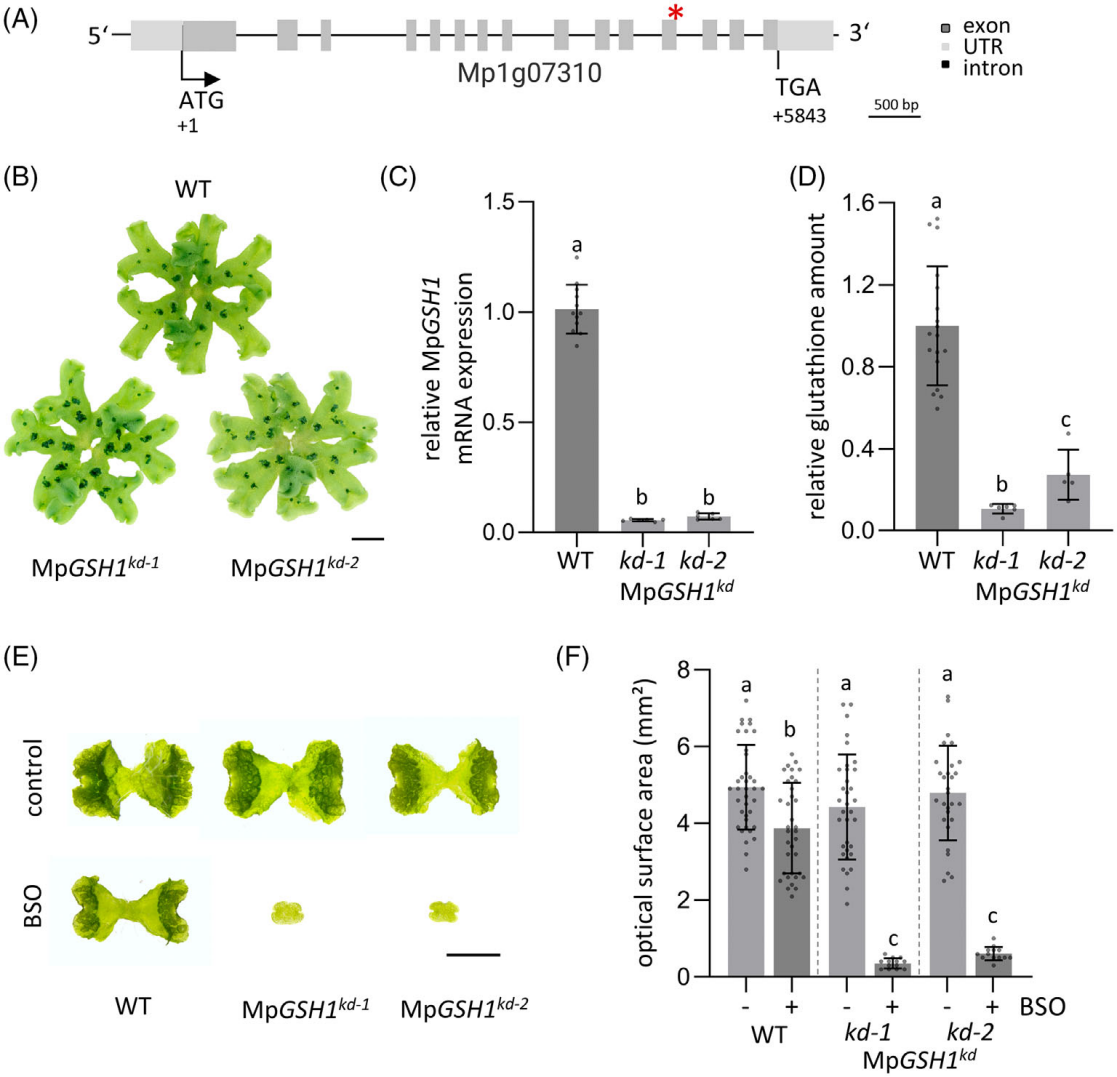
Mp*GSH1* knockdown mutant analysis reveals a crucial role of GSH biosynthesis for *Marchantia polymorpha* development. (A) Schematic representation of the Mp*GSH1* locus (Mp1g07310), highlighting exons as gray boxes, untranslated regions (UTRs) as light gray boxes, and introns as black lines. The red asterisk indicates the miRNA target site. (B) Phenotype of 28 DAG WT and Mp*GSH1* ^*kd*^ plants. Scale bar: 1 cm. (C) Relative Mp*GSH1* mRNA expression levels in 28 DAG Mp*GSH1* ^*kd*^ plants compared to WT plants determined by qRT‐PCR (*n* ≥ 6). (D) Quantification of total glutathione content in 28 DAG plants using the DTNB recycling assay (*n* ≥ 5). (E) Comparison of 7 DAG WT and Mp*GSH1* ^*kd*^ plants grown in the absence (control) or presence of 500 μM BSO. Scale bar: 2 mm. (F) Optical surface area quantification of 7 DAG WT and Mp*GSH1* ^*kd*^ plants, without (−) and with (+) 500 μM BSO treatment (*n* ≥ 13). Letters indicate statistically significant results (C, D, F). Data are represented as mean ± standard deviation. Statistical significance was tested using the single ANOVA followed by Tukey (*P* < 0.05).

To further investigate the role of GSH in regulating redox homeostasis and meristematic activities in *M. polymorpha*, knockdown mutant plants of the sole *GAMMA GLUTAMYLCYSTEINE SYNTHETASE* gene, Mp*GSH1* (Mp1g07310), were generated. In *A. thaliana*, *GSH1* is essential for GSH synthesis and indispensable, as no vital *AtGSH1* knockout mutants could be generated (Cairns et al., 2006). Similarly, several attempts using different guide RNAs (Figure S2a) failed to generate viable Mp*gsh1* CRISPR/Cas9 knockout plants, which underlines the importance of GSH1 functionality also in *M. polymorpha*. Therefore, an artificial microRNA knockdown approach was conducted as described by Flores‐Sandoval et al. (2016) to examine the effects of reduced Mp*GSH1* expression levels (Figure 2A). Fifteen Mp*GSH1* ^*kd*^ T1 lines were generated and analyzed by qRT‐PCR, all of which showed no recognizable aberrant phenotype under standard growth conditions (Figure 2B). One male (Mp*GSH1* ^*kd‐1*^) and one female (Mp*GSH1* ^*kd‐2*^) line were selected with the lowest residual Mp*GSH1* expression levels of 5% and 7%, respectively (Figure 2C). Next, the total GSH levels were analyzed by conducting a DTNB recycling assay (Salbitani et al., 2017; Tietze, 1969). Compared to WT plants, the GSH content was reduced to 11% in Mp*GSH1* ^*kd‐1*^ and to 27% in Mp*GSH1* ^*kd‐2*^ plants (Figure 2D), correlating with the respective Mp*GSH1* expression levels. Notably, although a complete Mp*gsh1* knockout is likely lethal, these low GSH levels in the knockdown mutants were still sufficient to maintain normal, WT‐like thallus growth. We compared the effect of further decreasing GSH levels with BSO between WT and knockdown mutants (Meyer et al., 2007). Seven days of 500 μM BSO treatment caused mild effects in WT plants that formed a slightly reduced thallus area. Contrarily, Mp*GSH1* ^*kd*^ gemmae development was severely affected, and both knockdown mutants showed a growth arrest (Figure 2E,F). Taken together, these data demonstrate a crucial role of GSH in *M. polymorpha* development. A further reduction in the knockdown mutants to below 11% of the total GSH level leads to severe growth defects in germinating gemmae (Figure S2b). The detected differences of the roGFP2‐hGrx1 oxidation levels between the meristem and surrounding differentiated cells in WT plants raised the question about spatial roGFP2‐hGrx1 oxidation dynamics in Mp*GSH1* knockdown and BSO‐treated mutant plants.

### In vivo roGFP2‐hGrx1 sensor analysis in WT and Mp*GSH1* ^*kd*^ plants

**Figure 3:**
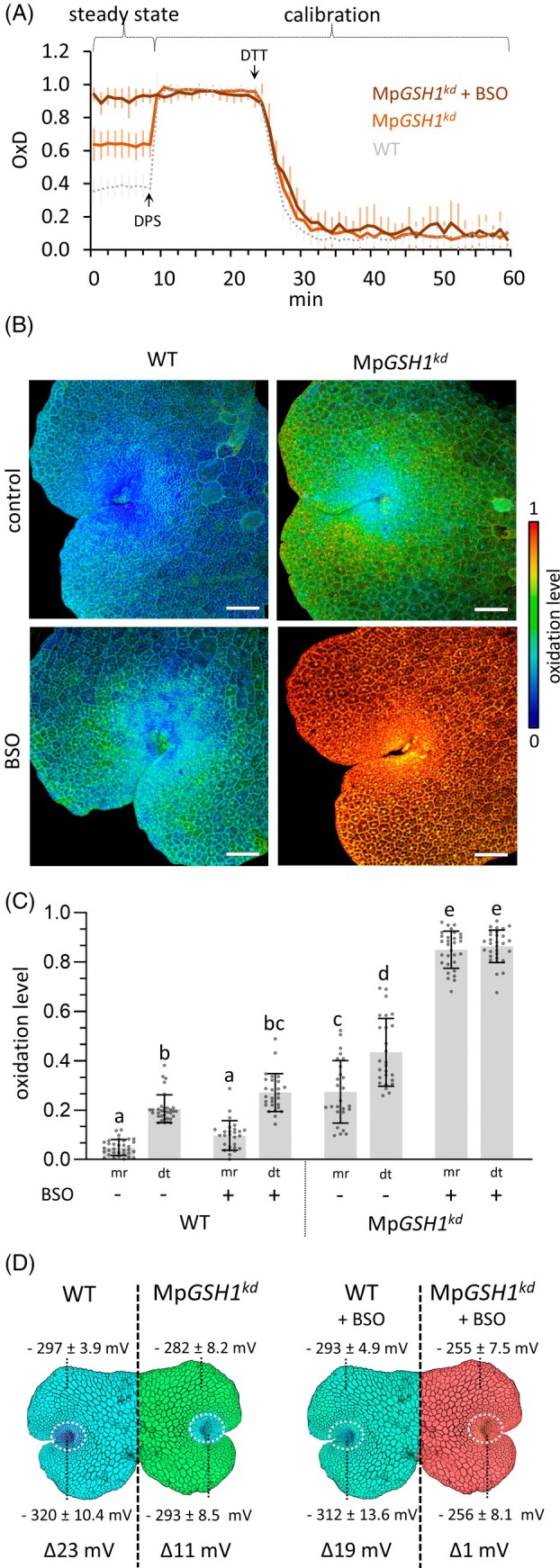
Redox dynamics in WT and Mp*GSH1* ^*kd*^ plants and effects of BSO treatment. (A) Plate reader measurements of roGFP2‐hGrx1 fluorescence in Mp*GSH1* ^*kd*^ plants. Gemmae were cultivated for 3 days on ½ GB medium and medium supplemented with 500 μM BSO. Fluorescence intensity was calibrated using 2 mM 2,2′‐dipyridyldisulfide (DPS) for oxidation and 20 mM dithiothreitol (DTT) for reduction. One male and one female line were analyzed (*n* = 9). (B) Ratiometric roGFP2‐hGrx1 fluorescence microscopy of 3 DAG *M. polymorpha* WT and Mp*GSH1* ^*kd*^ plants grown on control medium or medium supplemented with 100 μM BSO. Rainbow false colors represent the sensor redox state, with red indicating more oxidized and blue more reduced states. Scale bar: 50 μm. Representative microscopy images from analysis of over 10 plants per condition. (C) Quantitative roGFP2‐hGrx1 fluorescence analysis using normalized roGFP2‐hGrx1 ratios from the meristematic region (mr) and differentiated tissue (dt) from 3 DAG gemmae. Mean values ± SD (standard deviation) were calculated from *n* > 10 plants/condition. Statistical significance was determined using the single ANOVA followed by Tukey (*P* < 0.05). (D) Schematic representation of 3 DAG gemmae indicating calculated redox potentials (mV) for meristematic and differentiated regions and the determined redox gradients (Δ) under control and BSO treatment conditions.

Next, the roGFP2‐hGrx1 sensor was introduced into the two characterized Mp*GSH1* ^*kd*^ lines by gemma transformation. For each knockdown mutant, one transgenic line expressing roGFP2‐hGrx1 was selected for further analyses. Plate reader measurements in these Mp*GSH1* ^*kd*^ plants showed an increase in OxD to 0.64, almost twice as high as in the WT (OxD 0.37; Figure 3A). However, this change in the GSH redox homeostasis did not appear to affect gemma germination and growth (Figure 2E,F). To investigate the effects of further GSH level reduction, roGFP2‐hGrx1 Mp*GSH1* ^*kd*^ plants were treated with 500 μM BSO, which further increased the OxD to 0.95 (Figure 3A). Ratiometric microscopy was used to compare oxidation differences in roGFP2‐hGrx1 WT and roGFP2‐hGrx1 Mp*GSH1* ^*kd*^ plants under normal conditions and with BSO treatment. To determine redox changes with a high spatial resolution, multi‐stack images were analyzed by quantifying individual planes. The meristematic region of WT plants has a lower roGFP2‐hGrx1 oxidation level of 4% than the surrounding differentiated tissue with a sensor oxidation level of 20% (Figure 3B,C). In Mp*GSH1* ^*kd*^ lines (Figure 3C) the roGFP2‐hGrx1 oxidation levels were increased to 26% in the meristematic region and to 44% in differentiated tissues. Notably, although both Mp*GSH1* ^*kd*^ tissues show higher roGFP2‐hGrx1 oxidization levels compared to WT plants, the growth of GSH knockdown plants was not affected.

To elucidate the effects of a further reduction of cellular GSH levels, roGFP2‐hGrx1 WT and roGFP2‐hGrx1 Mp*GSH1* ^*kd*^ plants were cultivated for 3 days on medium supplemented with 100 μM BSO (Figure 3B,C). In WT plants, the roGFP2‐hGrx1 oxidation level increased to 10% in the meristematic region and to 26% in the differentiated thallus tissue. BSO‐treated Mp*GSH1* ^*kd*^ plants no longer showed a more reduced roGFP2‐hGrx1 state in the meristematic region; instead, over 90% of the roGFP2‐hGrx1 thiols were oxidized throughout the thallus (Figure 3C).

The GSH redox potential (*E*_GSH_) was determined by applying the Nernst equation adjusted for pH 7 and an experimental temperature of 23 °C (Aller et al., 2013; Schwarzländer et al., 2008). Accordingly, oxidation values of Figure 3C were converted into the redox potential in millivolts (mV). In WT plants, the meristematic region had an intracellular *E*_GSH_ of −320 ± 10 mV, while the *E*_GSH_ of the differentiated tissue was −297 ± 4 mV, resulting in a redox gradient of Δ23 mV between these two distinct tissues. In Mp*GSH1* ^*kd*^ plants, the meristematic region had an *E*_GSH_ of −293 ± 9 mV and the differentiated tissue an *E*_GSH_ of −282 ± 8 mV, forming a redox gradient of Δ11 mV. After treatment of WT plants with BSO, the *E*_GSH_ decreased in meristematic and differentiated tissues. However, a redox gradient of Δ19 mV was preserved between the two tissues. Conversely, further oxidation and the overall decrease of GSH levels in knockdown plants by treatment with BSO almost completely abolished the formation of a redox gradient and further growth (Figures 2E,F and 3D). These data demonstrate that the formation of an *E*_GSH_ gradient is crucial for the maintenance of meristem activities and growth processes in *M. polymorpha*.

### The HyPer7 sensor reports H_2_O_2_ dynamics in *M. polymorpha* meristems

As a noninvasive ratiometric fluorescent biosensing technique, the development of HyPer sensors facilitated the in vivo visualization of cellular H_2_O_2_ and measurements of physiological H_2_O_2_ levels in different mammalian tissues (Belousov et al., 2006; Pak et al., 2020) and *A. thaliana* (Ugalde, Schlößer, et al., 2021). HyPer7 consists of a circularly permutated yellow fluorescent protein fused with an OxyR‐RD domain derived from *Neisseria meningitidis* that sensitively reacts with H_2_O_2_. Following oxidation, HyPer7 forms an intramolecular disulfide bridge that alters the excitation spectra, enabling real‐time observations to determine H_2_O_2_ dynamics (Pak et al., 2020; Zhuravlev et al., 2024). Given the pivotal role of H_2_O_2_ in the regulation of meristem growth in *A. thaliana* (Tsukagoshi et al., 2010; Zeng et al., 2017), we applied this advanced pH‐insensitive sensor to investigate H_2_O_2_ gradients in *M. polymorpha* development. From over 10 transgenic T1 lines expressing the sensor under control of the Mp*EF1α* promoter, one male line and one female line exhibiting strong fluorescence were selected for further analyses.

**Figure 4:**
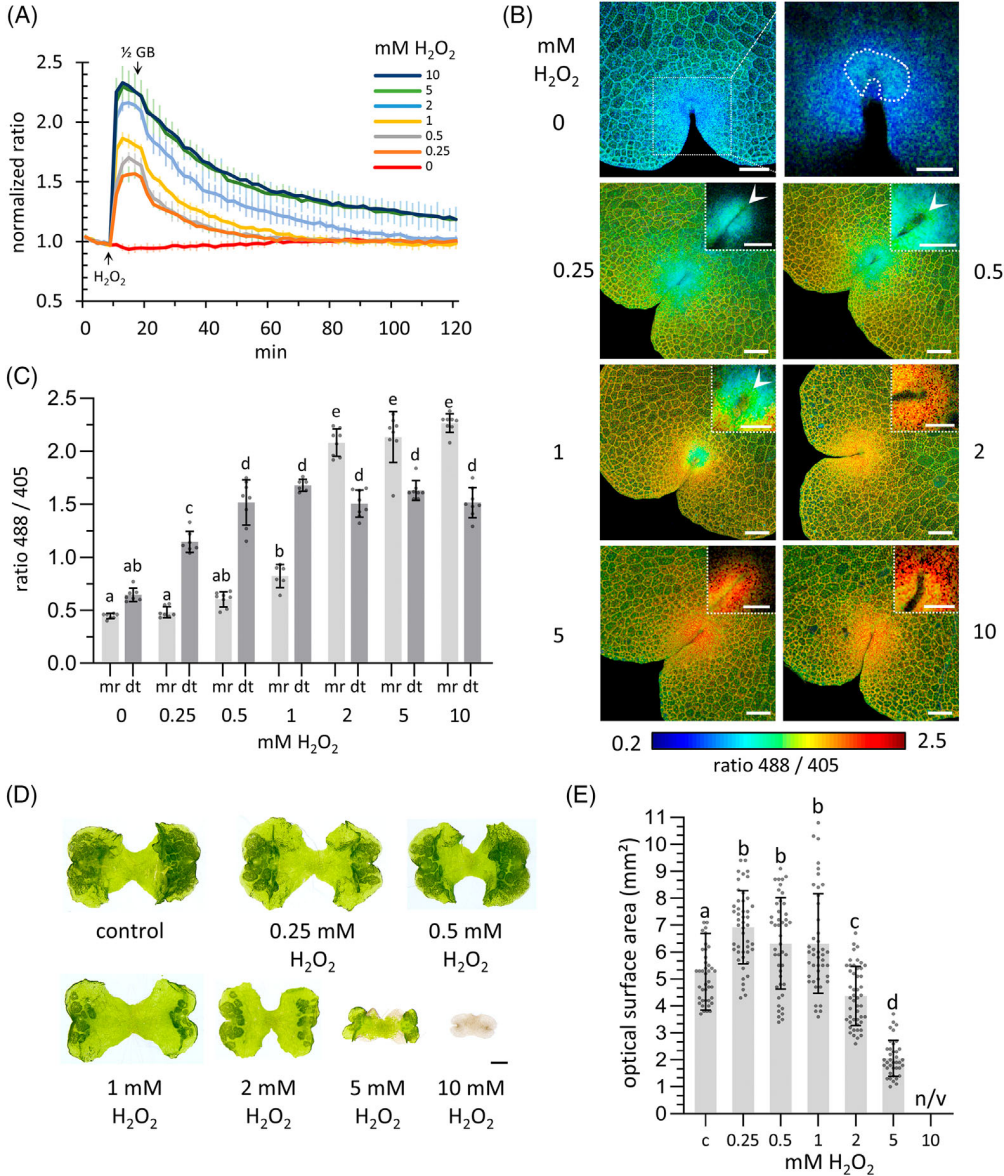
Functional characterization of HyPer7 as H_2_O_2_ sensor in 3 DAG gemmae. (A) Plate reader measurements of HyPer7 fluorescence intensity in 3 DAG gemmae. After 10 min, gemmae were submersed in medium with different H_2_O_2_ concentrations (0.25–10 mM) for 10 min, followed by recovery measurements for 100 min. Error bars represent standard deviation, calculated from *n* ≥ 6. Values were normalized to the 488/405 ratio of the first time point. (B) Microscopic images of HyPer7 fluorescence in 3 DAG transgenic gemmae exposed to the H_2_O_2_ series. The rainbow color scheme depicts the detected HyPer7 ratio 488/405 range from low (blue) to high (red) H_2_O_2_ concentrations. Ratiometric images visualize spectral changes in response to increasing H_2_O_2_ concentrations (*n* > 7). Scale bars, overview: 100 μm, insets: 50 μm. Images are maximum intensity *Z*‐stack projections processed using RRA software. Insets represent single slice images of corresponding overview *Z*‐stack projections. Arrows indicate higher oxidized meristematic zones, disappearing after 2 mM treatments. (C) HyPer7 ratios from the meristematic region (mr) and surrounding differentiated tissue (dt) were determined using representative images (*n* = 7) shown in (B). (D) Phenotypic analysis of WT plants after cultivation for 3 days, followed by 10 min H₂O₂ treatment and recovery for 4 days. Plants treated with 10 mM H₂O₂ were non‐viable and excluded from further analysis. Scale bar: 500 μm. (E) Quantitative assessment of the optical surface area of 7‐day‐old WT plants after H₂O₂ treatments (*n* > 35). Data are represented as mean ± standard deviation. Statistical significance was determined using the single ANOVA test followed by Tukey (*P* < 0.05).

First, the sensitivity of HyPer7 toward H_2_O_2_ treatment of 3 DAG gemmae was assessed by plate reader measurements (Figure 4A). After a 10 min measurement, HyPer7‐expressing plants were treated for 10 min with six different H_2_O_2_ concentrations ranging from 0.25 to 10 mM. Then, plants were monitored for 100 min to investigate the sensor recovery. HyPer7 showed a concentration‐dependent response to the H_2_O_2_ exposure. Plants treated with higher H_2_O_2_ concentrations of 5 and 10 mM failed to reach pretreatment HyPer7 ratios, and the H_2_O_2_ buffering capacity was thus exceeded under these conditions.

The Hyper7 sensor was localized in the cytosol and nuclei (Figure S1b). Next, we used the sensor for in vivo microscopy to record H_2_O_2_ levels with an enhanced spatial tissue resolution in HyPer7‐expressing gemmae that were exposed to the different H_2_O_2_ concentrations for 10 min (Figure 4B).

An uneven distribution of sensor oxidation was detected in young gemmae (Figure 4B). In the meristematic region, low oxidation of HyPer7 indicates low H_2_O_2_ levels in this area with high cell division activity. In contrast, surrounding tissues with differentiated cells exhibited higher H_2_O_2_ levels (Figure 4B). Further analysis of individual planes from ratiometric *Z*‐stack images unveiled the formation of a small central zone with increased HyPer7 sensor oxidation that is localized within the meristematic region with an overall higher degree of reduction (Figure 4B, insets). After 0.25 mM H_2_O_2_ treatment, the meristematic region maintained a more reduced sensor oxidation state, which increased in surrounding tissues with differentiated cells (Figure 4B,C). Further increases in H_2_O_2_ concentrations up to 1 mM raised oxidized HyPer7 levels in the small oxidation zone as well as in the cell differentiation zones (Figure 4B, insets). Finally, at 2 mM and higher H_2_O_2_ concentrations, the degree of HyPer7 oxidation in meristematic regions surpassed the levels determined for the surrounding differentiated tissue.

To investigate the impact of H_2_O_2_ on *M. polymorpha* growth, 3 DAG WT gemmae were exposed to different H_2_O_2_ concentrations for 10 min, followed by a 4‐day recovery period (Figure 4D,E). Treatment with 0.25–1 mM H_2_O_2_ concentrations resulted in enhanced gemmae growth compared to untreated controls (Figure 4E). In contrast, growth was significantly reduced in gemmae treated with higher concentrations of 2 and 5 mM H_2_O_2_. Exposure to 10 mM H_2_O_2_ inhibited further growth and led to plant death. Together, these data demonstrate the participation of quantitative and spatial H_2_O_2_ dynamics in the regulation of cell division and differentiation processes in *M. polymorpha*.

### MpCLE2p affects redox buffer and oxidative dynamics in meristematic regions

To corroborate these observations, we took advantage of the peptide MpCLE2 (MpCLE2p), which positively regulates the size of the stem cell zone in the *M. polymorpha* ecotype TAK (Hirakawa et al., 2020). In contrast to *A. thaliana*, where CLAVATA3 (CLV3) peptides reduce stem cell numbers, MpCLE2 is a member of the CLV3 peptide signaling family that positively regulates stem cell population size in the *M. polymorpha* meristem (Hirakawa et al., 2020; Takahashi et al., 2021).

**Figure 5:**
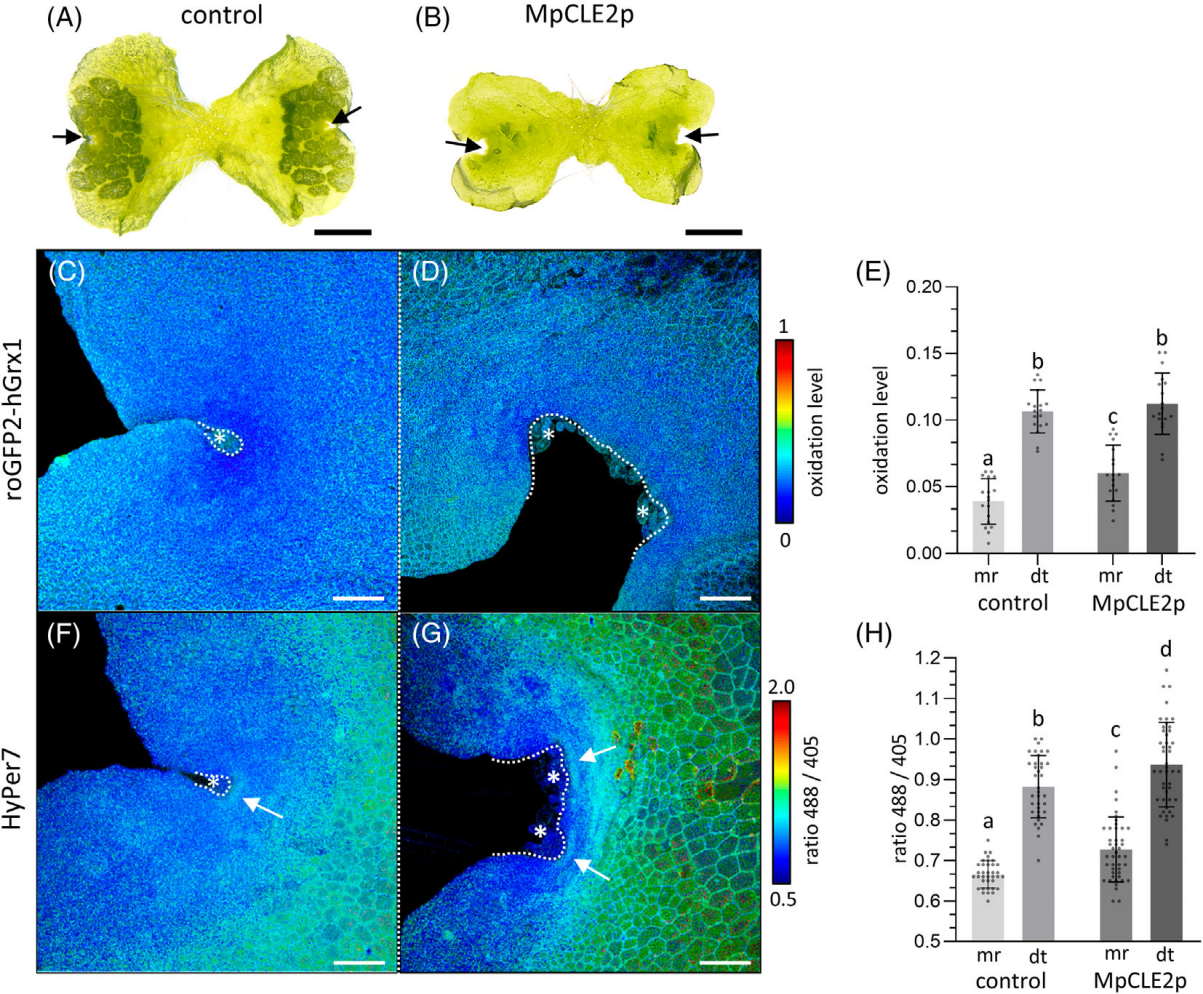
Monitoring of roGFP2‐hGrx1 and HyPer7 responses upon MpCLE2p treatment. (A) Morphology of a *M. polymorpha* control plant and (B) a plant after 7 days of 3 μM MpCLE2p treatment. Three male WT lines with at least five biological and three technical replicates per line were analyzed. Arrows indicate the meristematic region, which is enlarged after the peptide treatment. Scale bars: 1 mm. (C, D) RoGFP2‐hGrx1 fluorescence of a 7‐day‐old control plant (C) and of a plant grown on medium with 3 μM MpCLE2p, forming an expanded meristematic region (D). Scale bars: 100 μm. (E) Quantification of roGFP2‐hGrx1 oxidation levels in the meristematic region (mr) and differentiated tissue (dt) of control and MpCLE2p‐treated plants (*n* > 18). (F, G) Ratiometric microscopy of HyPer7 fluorescence of untreated (F) and MpCLE2p‐treated (G) plants. (H) Quantification of HyPer7 oxidation levels (*n* > 28). Dotted lines delineate the central meristem epidermis, where mucilage papillae (*) are formed that protect stem cells. Data are represented as mean ± standard deviation. Statistical significance was determined using the single ANOVA test followed by Tukey (*P* < 0.05).

As observed for the TAK ecotype, application of MpCLE2p to BoGa ecotype gemmae caused expansion of the meristematic region (Figure 5A,B). In the TAK ecotype, meristem expansion was described for 4 DAG gemmae. For the BoGa ecotype, an expansion was clearly visible in 7 DAG gemmae that were therefore used for further sensor analyses. Compared to the surrounding differentiated tissue, the meristematic region still had a more reduced GSH oxidation level, which is, however, significantly increased compared to untreated plants (Figure 5C–E). Next, MpCLE2p effects on H_2_O_2_ were investigated. Similar to 3 DAG plants (Figure 4B,C), 7 DAG HyPer7 control plants exhibited lower levels of sensor oxidation in the meristematic regions compared to differentiated cells (Figure 5F,H). Both ratios were elevated in MpCLE2p treated plants, but overall, the HyPer7 sensor was still more reduced in the meristematic region compared to the differentiated tissue (Figure 5G,H). Furthermore, as observed before for 3 DAG, analysis of single *Z*‐stack planes revealed a zone with higher H_2_O_2_ accumulation, which appeared in 7 DAG control plants as a more focused area encompassing the central ridge of the meristematic region (Figures 4B and 5F). Notably, we detected several elongated zones with higher HyPer7 oxidation in the expanded meristem of MpCLE2p‐treated plants (Figure 5G). Movies with 3D reconstructions visualize the oxidation pattern with an increased resolution (Figure S3), strengthening a distinctive accumulation of H_2_O_2_ in the enlarged meristematic region of MpCLE2p treated plants.

Together, application of MpCLE2p in transgenic plants expressing the roGFP2‐hGrx1 and HyPer7 sensors revealed a significant increase in *E*_GSH_ and H_2_O_2_ levels in the expanded meristematic region with an enhanced number of stem cells (Hirakawa et al., 2020) and in differentiated tissues. MpCLE2p treatment thus affects the meristematic ROS balance, corroborating the interplay of oxidation dynamics and regulation of cell proliferation in *M. polymorpha*.

## DISCUSSION

Plant growth is realized by meristems, whose anatomy diversified during the evolution of land plants (Arnoux‐Courseaux & Coudert, 2024; Fouracre & Harrison, 2022). In *A. thaliana*, a threshold level of the redox buffer GSH is required for meristem and further growth regulation (Noctor et al., 2024). Additionally, distinct ROS exhibit different regulatory activities in the *A. thaliana* SAM and RAM, where O_2_^•−^ maintains stemness and cell proliferation, whereas H_2_O_2_ promotes differentiation processes (Tsukagoshi et al., 2010; Zeng et al., 2017). Despite compelling evidence for crucial regulatory redox functions in developmental processes of angiosperms, knowledge about the participation of the GSH redox buffer and ROS in bryophytes, which form less complex meristems, is scarce. H_2_O_2_ is the most stable ROS and is mainly formed via dismutation of O_2_^•−^ by superoxide dismutases (SODs) and enzymatically scavenged by catalases (CATs), peroxidases, and peroxiredoxins (Mattila et al., 2015). In plants, a widely used chemical for H_2_O_2_ detection is DAB (3,3′‐diaminobenzidine), which leads to the formation of a brown precipitate but might also detect other ROS species. Another drawback shared with different fluorescent dyes is the generation of accumulating signals preventing analysis of in vivo ROS dynamics (Fichman et al., 2019). In the last decades, genetically encoded probes have extended the opportunities for in vivo monitoring of oxidative real‐time signals in plants (Aller et al., 2013; Dopp et al., 2023; Marty et al., 2009; Nietzel et al., 2018). Here, we employed the advanced redox sensors roGFP2‐hGrx1 and HyPer7 for an in vivo analysis in *M. polymorpha* that enabled an unprecedented spatial resolution of the GSH redox buffer and H_2_O_2_ activities and revealed crucial functions in cell division and differentiation processes.

### An *E*_GSH_ gradient contributes to growth regulation in *M. polymorpha*

The tripeptide GSH is an important antioxidant that confers redox stability to cells and participates in stress and developmental processes. Functionality of the roGFP2‐hGrx1 sensor as an *E*_GSH_ probe for in vivo imaging was demonstrated by using DPS and DTT for sensor oxidation and reduction, respectively. Treatment with the GSH synthesis inhibitor BSO showed a rapid increase in the OxD and impaired plant growth. The failure to generate Mp*gsh1* knockout mutants strengthens the importance of the GSH buffer for developmental processes in *M. polymorpha*. Viable Mp*GSH1* knockdown mutants indicate that GSH levels of at least 11% compared to WT are sufficient for normal *M. polymorpha* development, and further GSH reduction by BSO treatment then arrests growth. In *A. thaliana*, several allelic *GSH1* mutations variably decrease GSH and together suggest that slightly higher GSH levels of 20%–30% effectively support meristematic activities and vegetative growth in *A. thaliana* (Noctor et al., 2024; Shanmugam et al., 2012; Vernoux et al., 2000).

The high tissue resolution of this *M. polymorpha* analysis unveiled the formation of a Δ23 mV redox gradient in WT plants, with a more reduced state in the proliferating meristematic region (−320 mV) compared to more oxidized differentiated tissues (−297 mV). Although Mp*GSH1* ^*kd*^ plants displayed elevated levels of oxidation, they maintained a Δ11 mV redox gradient within the meristem. Further lowering of GSH levels by BSO treatment abrogated the formation of a gradient. Then, the whole thallus tissue was strongly oxidized, which led to growth arrest in Mp*GSH1* ^*kd*^ plants. The data indicate that the maintenance of a Δ11 mV gradient is crucial and sufficient for meristem vitality and normal growth.

The *E*_GSH_ of 3 and 7 DAG WT *M. polymorpha* plants is close to the *E*_GSH_ (−320 mV to −310 mV) determined for *A. thaliana* (Aller et al., 2013; Meyer et al., 2007; Schwarzländer et al., 2008), where *E*_GSH_ analyses have thus far mainly focused on responses to abiotic stressors such as oxygen deprivation, heat, heavy metal stress, or herbicide treatment (Schwarzländer et al., 2008; Ugalde, Fuchs, et al., 2021; Ugalde, Schlößer, et al., 2021; Wagner et al., 2019). One study with increased spatial resolution by Jiang et al. (2016) detected a similar redox gradient between the *A. thaliana* root meristem (−317 mV) and elongation zone (−300 mV), suggesting that redox gradient formation is crucial in land plants for the control of cell proliferation and differentiation processes.

### H_2_O_2_ dynamics coordinate growth processes in *M. polymorpha*

We generated H_2_O_2_‐sensitive HyPer7 *M. polymorpha* reporter lines, and plate reader measurements demonstrated the applicability of the biosensor to monitor in vivo oxidation dynamics of this ROS. After 0.25 mM up to 10 mM H_2_O_2_ treatments, normalized ratios decreased without addition of further reductants, revealing that functional reducing systems, such as the GSH redox buffer, likely contribute to compensate oxidative stress in *M. polymorpha*. Confocal in vivo microscopy detected spatial and quantitative H_2_O_2_ oxidation differences. In WT plants, the HyPer7 sensor is more reduced in the meristematic region and more oxidized in the surrounding differentiated tissue. After 0.25 to 1 mM H_2_O_2_ treatments of 3 DAG gemmae, the sensor sustained a more reduced state in the meristematic region, whereas the HyPer7 ratio increased in differentiated tissues, showing a stronger H_2_O_2_ accumulation. Single‐plane analysis in 3 DAG HyPer7 plants demonstrated the formation of a small, oxidized zone in the overall more reduced juvenile meristematic region. This oxidized zone was detectable in treatments up to 1 mM H_2_O_2_. At higher H_2_O_2_ concentrations, the whole meristematic zone was more oxidized, and buffering capacities were thus likely exploited, resulting in reduced plant growth and finally plant death. These findings highlight the importance of a more oxidized zone in the overall reduced meristematic region, formed during *M. polymorpha* meristem maturation and likely encompassing the area of stem cells and their proliferating merophyte derivatives. Furthermore, a non‐linear correlation between the H_2_O_2_ dose and growth effects was observed. Plant growth was enhanced by 0.25–1 mM H_2_O_2_, while an opposing effect was observed at higher H_2_O_2_ doses, finally causing cell death after 10 mM H_2_O_2_ treatments. In Capsicum, low doses of H_2_O_2_ were shown to stimulate development and growth of this crop, whereas higher doses exert negative effects (Angole‐Tierrablanca et al., 2025). Hormesis, the dose–response phenomenon to stressor application, has been observed in numerous organisms including angiosperms (Jalal et al., 2021; Salinitro et al., 2021) and likely also functions as an adaptive response to different stressor levels in the liverwort *M. polymorpha*. The pivotal role of H_2_O_2_ in growth regulation of *M. polymorpha* was further corroborated by combining HyPer7 and roGFP2‐hGrx1 microscopy with the application of the stem cell regulatory peptide MpCLE2p (Hirakawa et al., 2020). MpCLE2p treatment resulted in the formation of an enlarged, over‐proliferating meristem with an increased number of oxidized zones demonstrating H_2_O_2_ accumulation. Together, our data reveal the importance of tightly regulated H_2_O_2_ and GSH buffer levels, acting together in the regulation of plant growth in *M. polymorpha*.

A crucial role of ROS for *M. polymorpha* growth processes has been demonstrated by analysis of the bHLH TF Mp*TCP1*. Mp*tcp1* mutants exhibit severely enhanced H_2_O_2_ levels and a strongly reduced growth, a correlation also observed for the Mp*PLT* TF (Busch et al., 2019; Fu et al., 2024). H_2_O_2_ can react with many proteins by targeting exposed Cys residues of TF, receptors, channels, and metabolic enzymes, thereby causing thiol‐based oxidative posttranslational modifications (oxi‐PTM) that alter their structure and function (Foyer, 2018; Jones & Sies, 2015; Noctor et al., 2018; Zhou et al., 2023). MpTCP1 contains a single Cys, Cys131, that mediates redox‐dependent in vitro DNA binding, resulting in a loss of binding under oxidizing conditions. Thereby, MpTCP1 can likely respond via Cys‐based oxi‐PTM to redox changes and modulate transcriptional responses (Busch et al., 2019). Moreover, MpTCP1 regulates the expression of a comprehensive downstream redox network, comprising enzymes involved in H_2_O_2_ metabolism and transport. The largest deregulated group of genes in Mp*tcp1* plants is class III peroxidases (PRXIII) that function in the extracellular generation as well as removal of H_2_O_2_ and were identified as a strongly expanded family with developmental and adaptive evolutionary features (Beaulieu et al., 2025; Busch et al., 2019; Kidwai et al., 2020). Additionally, nine aquaporins, facilitating H_2_O_2_ transport from the apoplast across the cell membrane into the cytosol, were strongly upregulated in Mp*tcp1* plants, suggesting that an altered transport of H_2_O_2_ between apoplast and cytosol contributes to the increased levels of this ROS (Busch et al., 2019; Mukherjee et al., 2024). These findings strengthen the impact of redox processes in the regulation of TF activities and downstream networks that govern growth processes in *M. polymorpha*.

### Outlook

Our data demonstrate the crucial role of distinct *E*_GSH_ in the meristematic region and differentiated tissue of *M. polymorpha* that establish a redox gradient required for the coordination of cell proliferation and differentiation processes. The redox state of the GSH pool influences many processes as it equilibrates with all accessible protein thiols and is coupled to ROS scavenging and damage repair enzymatically via reactions such as the ascorbate‐glutathione cycle in plants (Mittler & Jones, 2024; Rahantaniaina et al., 2013). By linking H_2_O_2_ levels and protein redox states in cells, *E*_GSH_ buffers H_2_O_2_ fluctuations and redox states within cells (Mittler & Jones, 2024). During *A. thaliana* development, distinctive spatial and temporal expression of respiratory burst oxidase homologs and SODs generating O_2_^•−^ and H_2_O_2_, respectively, as well as PRX and CATs removing H_2_O_2_, together set H_2_O_2_ levels in differentiated tissues (Tsukagoshi et al., 2010; Zeng et al., 2017). Recent investigations of SODs in *M. polymorpha* further underline the importance of H_2_O_2_ for developmental, non‐stress ROS signaling in plant growth (Frohn et al., 2024). In *M. polymorpha*, increased H_2_O_2_ levels in the central, proliferating zone in the meristematic region differ from the situation in *A. thaliana*, where H_2_O_2_ accumulates in differentiating cells (Tsukagoshi et al., 2010; Zeng et al., 2017). In bryophytes and angiosperms, meristem functions are, at least in part, regulated via conserved pathways and molecular networks. However, while CLV signaling in *M. polymorpha* promotes stem cell activity, it exerts inhibitory functions in the SAM of *A. thaliana* and operates in a feedback loop with the TF WUS, which is negatively regulated by H_2_O_2_ (Hirakawa et al., 2019; Hirakawa et al., 2020; Schoof et al., 2000; Zeng et al., 2017). The WUS subfamily, belonging to the large WOX TF family, is absent in *M. polymorpha* and emerged in the ancestor of ferns and seed plants (Nardmann & Werr, 2012), emphasizing the importance of gene duplications followed by sub‐ and neofunctionalization. Meristem diversification was driven by the rewiring of molecular regulatory modules and co‐option of genes (Arnoux‐Courseaux & Coudert, 2024). Our data suggest that differences in H_2_O_2_ levels provide an additional mechanism to modulate regulatory network activities that contributed to increased anatomical meristem complexity and plant growth. It will thus be interesting to understand the contribution of more players to establish the dynamics of different ROS types including O_2_^•−^, downstream oxi‐PTM targets and the intertwined relationship to the GSH buffer in developmental processes. Comparative in vivo redox/ROS sensor analyses in diverse bryophyte and vascular plants can unveil their functions to cope with changing environmental conditions and unveil potential implications for agriculture.

## EXPERIMENTAL PROCEDURES

### Plants and growing conditions

*Marchantia polymorpha ssp. ruderalis* plants (ecotype BoGa from the Botanical Garden of the Osnabrück University, Germany) were cultivated on solid ½ Gamborg B5 medium containing vitamins and 1.4% or 0.5% agar‐agar Kobe I under long‐day conditions (16 h light: 8 h darkness) at 22 °C and 80 μmol m^−2^ s^−1^ light intensity (Althoff et al., 2014). Plants were propagated vegetatively by transferring gemmae. Gemmae were examined 3 and 7 days after germination (DAG). For treatment with L‐buthionine‐(S,R)‐sulfoximine (BSO), plants were grown for 3–7 days on growth medium supplemented with 500 μM or 100 μM BSO, diluted in water (Cayman Chemicals). The MpCLE2 peptide KEVHypNGHypNPLHN (Hirakawa et al., 2020) was purchased from GenScript Biotech (Netherlands). For apical notch extension analyses, 3 μM MpCLE2p was added to the growth medium and gemmae were cultivated for 7 days. HyPer7 expressing lines were pre‐cultivated for 3 days prior to H_2_O_2_ treatment. Subsequently, the plants were exposed to H_2_O_2_ concentrations ranging from 0 to 10 mM for 10 min. Following treatment, plants were transferred back to cultivation plates and allowed to recover for an additional 4 days.

### Generation of transgenic *M. polymorpha* plants

A Gateway™ entry vector containing the CDS for the fusion protein roGFP2‐hGrx1 (Albrecht et al., 2014) was obtained from Andreas Meyer (Bonn). Valeriy V. Pak and Vsevolod V. Belousov (Moscow) kindly provided the CDS of HyPer7 (Pak et al., 2020), which was introduced into pDONR207™. For redox sensor analyses in *M. polymorpha*, roGFP2‐hGrx1 and HyPer7 sequences were then transferred into the pMpGWB403 destination vector (Ishizaki et al., 2016), comprising the Mp*EF1α* promoter driving ubiquitous expression (Althoff et al., 2014). The resulting destination vectors were employed for wildtype (WT) sporeling transformations according to Ishizaki et al. (2016) using *Agrobacterium tumefaciens* (strain C58C1 pGV2260). To express roGFP2‐hGrx1 in Mp*GSH1* ^*kd*^ plants, mutant gemmae were utilized for transformation. All primer sequences are listed in Table S1.

A CRISPR/Cas9 approach was carried out to induce a frameshift mutation in the Mp*GSH1* (Mp1g07310) locus. Five synthetic guide RNAs (Table S1) were generated. Cloning was conducted as described by Sugano et al. (2018) using pMpGE_En03 and pMpGE010. No viable Mp*gsh1* plants were generated. In consequence, an artificial miRNA knockdown approach was conducted to generate Mp*GSH1* knockdown lines. A target sequence with high specificity was selected and verified by BLAST analysis to exclude additional target regions (Flores‐Sandoval et al., 2016). The Mp*MIR160* precursor, *amiR‐*Mp*GSH1* ^*MpMIR160*^ with the target sequence (ATCCCTGCAAGGAGCGTTGGA) was synthesized by GenScript Biotech (Netherlands) and introduced into the entry vector pENTR™4 Dual Gateway™. After transfer into the destination vector pMpGWB103 (Ishizaki et al., 2016) comprising the Mp*EF1α* the construct was used in a sporeling transformation.

### Analysis of Mp*GSH1* ^*kd*^ plants

To select amiR‐Mp*GSH1* ^Mp*mir160*^ lines with reduced expression levels, 15 transgenic plants were analyzed in comparison to WT lines by qRT‐PCR. RNA isolation and further analysis were conducted as described by Busch et al. (2019). Mean normalized expression levels for each sample were calculated from three biological replicates, with Mp*EF1α* as the reference gene. One male (Mp*GSH1* ^*kd‐1*^) and one female line (Mp*GSH1* ^*kd‐2*^) were selected for further analysis.

To quantify the GSH amount, sample preparation of 4‐week‐old *M. polymorpha* thallus tissue and measurement preparation were carried out according to Queval and Noctor (2007) and Salbitani et al. (2017), originally based on the DTNB recycling assay of Tietze (1969). Quantification was executed with the SPECORD 50 spectrophotometer (Analytik Jena) over a period of 5 min, monitoring the absorption at 412 nm. The total GSH concentration of each sample was then calculated by comparing it to the slope of a GSH calibration curve.

The optical surface areas of 7 DAG WT and Mp*GSH1* ^*kd*^ plants were documented with a Canon EOS 750D camera. Calculations of the area were conducted with ImageJ2 (Fiji v.2.3.0/1.53f).

### Ratiometric microscopy

Ratiometric microscopy using specific biosensors, such as roGFP2‐hGrx1 and HyPer7, facilitates an analysis independent of fluorophore concentration. The biosensors employed for the determination of the GSH redox potential (*E*_GSH_) and visualization of intracellular H_2_O_2_ concentration (HyPer7) are ratiometric fluorophores (Meyer et al., 2007; Pak et al., 2020). Confocal imaging of *M. polymorpha* gemmae was conducted at 23 °C using a ZEISS LSM880 inverted confocal microscope equipped with a 405 nm diode laser and a 488 nm Ar‐Laser, utilizing a Plan‐Apochromat 20× (NA 0.8) objective. The recording area covered 708.5 × 708.5 μm and gemmae were submersed in ½ GB basal salt imaging medium. The programs Fiji and Redox Ratio Analysis (RRA) (Fricker, 2016) were applied to process acquired images. For the quantification of the ratiometric values of different tissues, an area of 100 × 100 μm was assessed. The ratiometric images are displayed in a rainbow false color scheme. False color images were saved as uncompressed .avi files and further processed using Fiji V.1.52n by maximum intensity *Z*‐stack projections, depending on specimen thickness, with each optical section measuring 4.69 μm. For the 3D reconstruction, the 3D Project Stack operation was conducted on the processed ratiometric images; the obtained movies were saved as a .avi file.

### Microscopic imaging of roGFP2‐hGrx1

Plants expressing roGFP2‐hGrx1 were sequentially excited at 405 and 488 nm, and the fluorescence emission was acquired at 500–550 nm. The pinhole was set to four airy units. Autofluorescence was collected after excitation at 405 nm, using an emission of 425–475 nm. The background was subtracted from the roGFP2‐hGrx1 images because plant cells exhibit autofluorescence after excitation at 405 nm that partially overlaps with roGFP2‐hGrx1 emission (Schwarzländer et al., 2008). A ratiometric analysis was performed by calculating the 405/488 nm ratio for each pixel using the Matlab‐based program Redox Ratio Analysis (RRA), resulting in a false color ratiometric image according to Fricker (2016). The resulting images are visualized by a rainbow false color table ranging from blue (minimal possible oxidation) to red (maximal possible oxidation).

Prior to each measurement, in vivo roGFP2‐hGrx1 calibration was conducted by imaging 5 gemmae treated with either 2 mM 2,2′‐dipyridyl disulfide (DPS) to achieve full oxidation or 20 mM dithiothreitol (DTT) to achieve complete reduction. The resulting 405/488 nm emission ratios were used for normalization, establishing a scale ranging from 0% (fully reduced) to 100% (fully oxidized) biosensor oxidation. This normalized scale was used to determine the oxidation level of the roGFP2‐hGrx1 biosensor in vivo. Subsequently, the *E*_GSH_ was calculated based on these oxidation levels using the Nernst equation adjusted for physiological conditions, specifically pH 7.0, an experimental temperature of 23 °C, and a consensus midpoint redox potential of roGFP2‐hGrx1 of −280 mV (Aller et al., 2013; Schwarzländer et al., 2008).

### Microscopic imaging of HyPer7

The H_2_O_2_ biosensor HyPer7 was used to visualize the tissue‐specific H_2_O_2_ levels in *M. polymorpha*. Oxidation by H_2_O_2_ alters the excitation spectra oppositely to roGFP2‐hGrx1, measurable in a ratiometric fashion (Pak et al., 2020).

Plant lines expressing HyPer7 underwent the same handling as roGFP2‐hGrx1 lines. Given the HyPer7 insensitivity to DTT, a DTT calibration cannot be conducted (Ugalde, Schlößer, et al., 2021). The GFP emission intensity ratio at 488 nm and 405 nm excitation was calculated from confocal image stacks. The 488/405 ratio quantitatively reflects the oxidation state of HyPer7, exhibiting a positive correlation with H_2_O_2_ concentration‐dependent oxidation. HyPer7 sensor oxidation is visualized using a rainbow false color scheme ranging from deep blue to red.

### Multiwell‐based fluorimetry

RoGFP2‐hGrx1 and HyPer7 plants underwent sequential excitation at 390 ± 10 nm and 480 ± 10 nm, with emission recorded at 520 ± 5 nm using the FLUOstar Omega plate reader. For each measurement, twenty 3 DAG or ten 7 DAG gemmae were filled in black 96‐well plates submerged in 100 μL ½ GB medium. RoGFP2‐hGrx1 calibration was performed using 2 mM DPS and 20 mM DTT. The degree of oxidation (OxD) of roGFP2‐hGrx1 was calculated according to Aller et al. (2013). RoGFP2‐hGrx1 measurements lasted 45 min, including 10 min for steady state, 15 min for oxidation, and 20 min for reduction. HyPer7 measurements were similarly conducted spanning 120 min, comprising 10 min for a steady‐state phase, 10 min for oxidation induction, and 100 min for recovery. The obtained data in HyPer7 measurements was calculated into a 488/405 nm ratio and normalized to the first measured values of each sample.

### Statistical evaluation

The statistical analysis of data obtained from this study was performed by PRISM10 (GraphPad Software, www.graphpad.com, San Diego, USA). Mean values were evaluated using analysis of variance (ANOVA), followed by a post hoc Tukey's test. Pairwise comparisons between two mean values were conducted via single ANOVA tests. Statistical significance was denoted by different lowercase letters when the *P*‐value was below 0.05. Data visualization was executed with GraphPadPrism10 or Excel.

## Author Contributions

CK, JH, SJMS, and SZ designed the research. CK, JH, NG, and TD performed the research. All authors analyzed the data. CK and SZ wrote the manuscript with support from all co‐authors, and all authors reviewed the manuscript.

## Conflict of Interest

The authors declare that they have no conflicts of interest associated with this work.

## Supporting information


**Figure S1.** Subcellular localization of the biosensors. (a) Subcellular localization of roGFP2‐hGrx1 fluorescence. (b) Subcellular localization of HyPer7 fluorescence. Scale bars 10 μm.
**Figure S2.** Visualization of the Mp*gsh1* knockout gRNAs and quantification of the total GSH amount after BSO treatment. (a) Positions of the five gRNAs (red circle) in the Mp*GSH1* gene locus for the CRISPR‐Cas9 knockout approach. (b) The total GSH content of 28 DAG plants grown with or without 500 μM BSO. The GSH level was quantified using the DTNB recycling assay (*n* ≥ 5). Data are represented as mean ± standard deviation. Statistical significance was determined using the single ANOVA test followed by Tukey (*P* < 0.05).
**Figure S3.** Detailed meristem analysis of the HyPer7 sensor in 7‐day‐old *M. polymorpha* plants. Movies visualize HyPer7 redox states in the meristematic region of *M. polymorpha* plants. (a) 3D reconstruction of a control plant, showing that a small zone with an oxidized HyPer7 state is localized in the center of an overall more reduced meristematic region. (b) 3D reconstruction of a plant treated with MpCLE2p, unveiling an increased number of expanded zones with higher HyPer7 oxidation formed in the extended and more reduced meristematic region. (c, d) Single‐plane movies for detailed observation of the oxidation dynamics within the meristematic region of control (c) and MpCLE2p‐treated (d) plants.

**Table S1.** Primer sequences used in this study.

**Video S1.** Video of Figure S3.

## Data Availability

All data generated or analyzed during this study are included in this article and its supplementary information.

## References

[tpj70317-bib-0001] Albrecht, S.C. , Sobotta, M.C. , Bausewein, D. , Aller, I. , Hell, R. , Dick, T.P. et al. (2014) Redesign of genetically encoded biosensors for monitoring mitochondrial redox status in a broad range of model eukaryotes. Journal of Biomolecular Screening, 19(3), 379–386. Available from: 10.1177/1087057113499634 23954927

[tpj70317-bib-0002] Ali, M.F. & Muday, G.K. (2024) Reactive oxygen species are signaling molecules that modulate plant reproduction. Plant, Cell & Environment, 47(5), 1592–1605. Available from: 10.1111/pce.14837 38282262

[tpj70317-bib-0003] Aller, I. , Rouhier, N. & Meyer, A.J. (2013) Development of roGFP2‐derived redox probes for measurement of the glutathione redox potential in the cytosol of severely glutathione‐deficient rml1 seedlings. Frontiers in Plant Science, 4, 506. Available from: 10.3389/fpls.2013.00506 24379821 PMC3863748

[tpj70317-bib-0004] Althoff, F. , Kopischke, S. , Zobell, O. , Ide, K. , Ishizaki, K. , Kohchi, T. et al. (2014) Comparison of the MpEF1alpha and CaMV35 promoters for application in Marchantia polymorpha overexpression studies. Transgenic Research, 23(2), 235–244. Available from: 10.1007/s11248-013-9746-z 24036909

[tpj70317-bib-0005] Angole‐Tierrablanca, J.A. , Jiménez‐Hernández, A. , Aguilar‐Rodríguez, P. , Feregrino‐Perez, A.A. , Rico‐Chávez, A.K. , Godínez‐Mendoza, P.L. et al. (2025) Hydrogen peroxide application based on a Hormetic scheme biostimulates L. grown under two fertigation regimes. Journal of Soil Science and Plant Nutrition, 25(1), 693–709. Available from: 10.1007/s42729-024-02161-6

[tpj70317-bib-0006] Arnoux‐Courseaux, M. & Coudert, Y. (2024) Re‐examining meristems through the lens of evo‐devo. Trends in Plant Science, 29(4), 413–427. Available from: 10.1016/j.tplants.2023.11.003 38040554

[tpj70317-bib-0007] Beaulieu, C. , Libourel, C. , Zamar, D.L.M. , El Mahboubi, K. , Hoey, D.J. , Greiff, G.R.L. et al. (2025) The pangenome reveals ancient mechanisms of plant adaptation to the environment. Nature Genetics, 57(3), 729–740. Available from: 10.1038/s41588-024-02071-4 39962240 PMC11906373

[tpj70317-bib-0008] Becker, A. , Chen, X. , Dresselhaus, T. , Gutsche, N. , Müller‐Schüssele, S. , Sprunck, S. et al. (2025) Sexual reproduction in land plants: an evolutionary perspective. Plant Reproduction, 38, 12. Available from: 10.1007/s00497-025-00522-4 40355640 PMC12069490

[tpj70317-bib-0009] Belousov, V.V. , Fradkov, A.F. , Lukyanov, K.A. , Staroverov, D.B. , Shakhbazov, K.S. , Terskikh, A.V. et al. (2006) Genetically encoded fluorescent indicator for intracellular hydrogen peroxide. Nature Methods, 3(4), 281–286. Available from: 10.1038/nmeth866 16554833

[tpj70317-bib-0010] Bowman, J.L. , Kohchi, T. , Yamato, K.T. , Jenkins, J. , Shu, S. , Ishizaki, K. et al. (2017) Insights into land plant evolution garnered from the Marchantia polymorpha genome. Cell, 171(2), 287–304.e215. Available from: 10.1016/j.cell.2017.09.030 28985561

[tpj70317-bib-0011] Busch, A. , Deckena, M. , Almeida‐Trapp, M. , Kopischke, S. , Kock, C. , Schussler, E. et al. (2019) MpTCP1 controls cell proliferation and redox processes in Marchantia polymorpha. The New Phytologist, 224(4), 1627–1641. Available from: 10.1111/nph.16132 31433873

[tpj70317-bib-0012] Cairns, N.G. , Pasternak, M. , Wachter, A. , Cobbett, C.S. & Meyer, A.J. (2006) Maturation of arabidopsis seeds is dependent on glutathione biosynthesis within the embryo. Plant Physiology, 141(2), 446–455. Available from: 10.1104/pp.106.077982 16531482 PMC1475471

[tpj70317-bib-0013] Considine, M.J. & Foyer, C.H. (2021) Oxygen and reactive oxygen species‐dependent regulation of plant growth and development. Plant Physiology, 186(1), 79–92. Available from: 10.1093/plphys/kiaa077 33793863 PMC8154071

[tpj70317-bib-0014] de Simone, A. , Hubbard, R. , de la Torre, N.V. , Velappan, Y. , Wilson, M. , Considine, M.J. et al. (2017) Redox changes during the cell cycle in the embryonic root meristem ofArabidopsis thaliana. Antioxidants & Redox Signaling, 27(18), 1505–1519. Available from: 10.1089/ars.2016.6959 28457165 PMC5678362

[tpj70317-bib-0015] de Vries, J. & Archibald, J.M. (2018) Plant evolution: landmarks on the path to terrestrial life. New Phytologist, 217(4), 1428–1434. Available from: 10.1111/nph.14975 29318635

[tpj70317-bib-0016] Dopp, I.J. , Kalac, K. & Mackenzie, S.A. (2023) Hydrogen peroxide sensor HyPer7 illuminates tissue‐specific plastid redox dynamics. Plant Physiology, 193(1), 217–228. Available from: 10.1093/plphys/kiad307 37226328 PMC10702466

[tpj70317-bib-0017] Fichman, Y. , Miller, G. & Mittler, R. (2019) Whole‐plant live imaging of reactive oxygen species. Molecular Plant, 12(9), 1203–1210. Available from: 10.1016/j.molp.2019.06.003 31220601

[tpj70317-bib-0018] Flores‐Sandoval, E. , Dierschke, T. , Fisher, T.J. & Bowman, J.L. (2016) Efficient and inducible use of artificial MicroRNAs inMarchantia polymorpha. Plant and Cell Physiology, 57(2), 281–290. Available from: 10.1093/pcp/pcv068 25971256

[tpj70317-bib-0019] Fouracre, J.P. & Harrison, C.J. (2022) How was apical growth regulated in the ancestral land plant? Insights from the development of non‐seed plants. Plant Physiology, 190(1), 100–112. Available from: 10.1093/plphys/kiac313 35771646 PMC9434304

[tpj70317-bib-0020] Foyer, C.H. (2018) Reactive oxygen species, oxidative signaling and the regulation of photosynthesis. Environmental and Experimental Botany, 154, 134–142. Available from: 10.1016/j.envexpbot.2018.05.003 30283160 PMC6105748

[tpj70317-bib-0021] Fricker, M.D. (2016) Quantitative redox imaging software. Antioxidants & Redox Signaling, 24(13), 752–762. Available from: 10.1089/ars.2015.6390 26154420

[tpj70317-bib-0022] Frohn, S. , Haas, F.B. , Chavez, B.G. , Dreyer, B.H. , Reiss, E.V. , Ziplys, A. et al. (2024) Evolutionary conserved and divergent responses to copper zinc superoxide dismutase inhibition in plants. Plant, Cell & Environment. Available from: 10.1111/pce.15198 39400938

[tpj70317-bib-0023] Fu, J. , Zhou, C. , Ma, F. , Zhao, J. , Yu, F. & Cui, H. (2024) The PLETHORA homolog in Marchantia polymorpha is essential to meristem maintenance, developmental progression, and redox homeostasis. Plant Cell Physiology, 65, 1231–1244. Available from: 10.1093/pcp/pcae055 38757817

[tpj70317-bib-0024] Gutscher, M. , Pauleau, A.L. , Marty, L. , Brach, T. , Wabnitz, G.H. , Samstag, Y. et al. (2008) Real‐time imaging of the intracellular glutathione redox potential. Nature Methods, 5(6), 553–559. Available from: 10.1038/Nmeth.1212 18469822

[tpj70317-bib-0025] Hanson, G.T. , Aggeler, R. , Oglesbee, D. , Cannon, M. , Capaldi, R.A. , Tsien, R.Y. et al. (2004) Investigating mitochondrial redox potential with redox‐sensitive green fluorescent protein indicators. Journal of Biological Chemistry, 279(13), 13044–13053. Available from: 10.1074/jbc.M312846200 14722062

[tpj70317-bib-0026] Harris, B.J. , Clark, J.W. , Schrempf, D. , Szöllosi, G.J. , Donoghue, P.C.J. , Hetherington, A.M. et al. (2022) Divergent evolutionary trajectories of bryophytes and tracheophytes from a complex common ancestor of land plants. Nature Ecology & Evolution, 6(11), 1634. Available from: 10.1038/s41559-022-01885-x 36175544 PMC9630106

[tpj70317-bib-0027] Hata, Y. & Kyozuka, J. (2021) Fundamental mechanisms of the stem cell regulation in land plants: lesson from shoot apical cells in bryophytes. Plant Molecular Biology, 107(4–5), 213–225. Available from: 10.1007/s11103-021-01126-y 33609252 PMC8648652

[tpj70317-bib-0028] Hirakawa, Y. , Fujimoto, T. , Ishida, S. , Uchida, N. , Sawa, S. , Kiyosue, T. et al. (2020) Induction of Multichotomous branching by CLAVATA peptide in Marchantia polymorpha. Current Biology, 30(19), 3833–3840.e3834. Available from: 10.1016/j.cub.2020.07.016 32822612

[tpj70317-bib-0029] Hirakawa, Y. , Uchida, N. , Yamaguchi, Y.L. , Tabata, R. , Ishida, S. , Ishizaki, K. et al. (2019) Control of proliferation in the haploid meristem by CLE peptide signaling in Marchantia polymorpha. PLoS Genetics, 15(3), e1007997. Available from: 10.1371/journal.pgen.1007997 30845139 PMC6424463

[tpj70317-bib-0030] Ishizaki, K. , Nishihama, R. , Yamato, K.T. & Kohchi, T. (2016) Molecular genetic tools and techniques forMarchantia polymorphaResearch. Plant and Cell Physiology, 57(2), 262–270. Available from: 10.1093/pcp/pcv097 26116421

[tpj70317-bib-0031] Jalal, A. , de Oliveira, J.J.r. , Ribeiro, J.S. , Fernandes, G.C. , Mariano, G.G. , Trindade, V.D.R. et al. (2021) Hormesis in plants: physiological and biochemical responses. Ecotoxicology and Environmental Safety, 207, 111225. Available from: 10.1016/j.ecoenv.2020.111225 32916526

[tpj70317-bib-0032] Jiang, K. , Moe‐Lange, J. , Hennet, L. & Feldman, L.J. (2016) Salt stress affects the redox status of Arabidopsis root meristems. Frontiers in Plant Science, 7, 81. Available from: 10.3389/fpls.2016.00081 26904053 PMC4744855

[tpj70317-bib-0033] Jones, D.P. & Sies, H. (2015) The redox code. Antioxidants & Redox Signaling, 23(9), 734–746. Available from: 10.1089/ars.2015.6247 25891126 PMC4580308

[tpj70317-bib-0034] Kidwai, M. , Ahmad, I.Z. & Chakrabarty, D. (2020) Class III peroxidase: an indispensable enzyme for biotic/abiotic stress tolerance and a potent candidate for crop improvement. Plant Cell Reports, 39(11), 1381–1393. Available from: 10.1007/s00299-020-02588-y 32886139

[tpj70317-bib-0035] Kohchi, T. , Yamato, K.T. , Ishizaki, K. , Yamaoka, S. & Nishihama, R. (2021) Development and molecular genetics of. Annual Review of Plant Biology, 72, 677–702. Available from: 10.1146/annurev-arplant-082520-094256 33684298

[tpj70317-bib-0036] Marty, L. , Siala, W. , Schwarzländer, M. , Fricker, M.D. , Wirtz, M. , Sweetlove, L.J. et al. (2009) The NADPH‐dependent thioredoxin system constitutes a functional backup for cytosolic glutathione reductase in. Proceedings of the National Academy of Sciences of the United States of America, 106(22), 9109–9114. Available from: 10.1073/pnas.0900206106 19451637 PMC2690020

[tpj70317-bib-0037] Mattila, H. , Khorobrykh, S. , Havurinne, V. & Tyystjärvi, E. (2015) Reactive oxygen species: reactions and detection from photosynthetic tissues. Journal of Photochemistry and Photobiology, B: Biology, 152, 176–214. Available from: 10.1016/j.jphotobiol.2015.10.001 26498710

[tpj70317-bib-0038] Meyer, A.J. , Brach, T. , Marty, L. , Kreye, S. , Rouhier, N. , Jacquot, J.‐P. et al. (2007) Redox‐sensitive GFP in Arabidopsis thalianais a quantitative biosensor for the redox potential of the cellular glutathione redox buffer. The Plant Journal, 52(5), 973–986. Available from: 10.1111/j.1365-313X.2007.03280.x 17892447

[tpj70317-bib-0039] Meyer, A.J. & Dick, T.P. (2010) Fluorescent protein‐based redox probes. Antioxidants & Redox Signaling, 13(5), 621–650. Available from: 10.1089/ars.2009.2948 20088706

[tpj70317-bib-0040] Mittler, R. (2017) ROS are good. Trends in Plant Science, 22(1), 11–19. Available from: 10.1016/j.tplants.2016.08.002 27666517

[tpj70317-bib-0041] Mittler, R. & Jones, D.P. (2024) The redox code of plants. Plant, Cell & Environment, 47(8), 2821–2829. Available from: 10.1111/pce.14787 38088476

[tpj70317-bib-0042] Mittler, R. , Zandalinas, S.I. , Fichman, Y. & Van Breusegem, F. (2022) Reactive oxygen species signalling in plant stress responses. Nature Reviews Molecular Cell Biology, 23(10), 663–679. Available from: 10.1038/s41580-022-00499-2 35760900

[tpj70317-bib-0043] Moubayidin, L. & Ostergaard, L. (2015) Symmetry matters. New Phytologist, 207(4), 985–990. Available from: 10.1111/nph.13526 26086581

[tpj70317-bib-0044] Mukherjee, S. , Roy, S. & Corpas, F.J. (2024) Aquaporins: a vital nexus in H_2_O_2_‐gasotransmitter signaling. Trends in Plant Science, 29(6), 681–693. Available from: 10.1016/j.tplants.2023.11.021 38199830

[tpj70317-bib-0045] Müller‐Schüssele, S.J. , Bohle, F. , Rossi, J. , Trost, P. , Meyer, A.J. & Zaffagnini, M. (2021) Plasticity in plastid redox networks: evolution of glutathione‐dependent redox cascades and glutathionylation sites. BMC Plant Biology, 21(1), 322. Available from: 10.1186/s12870-021-03087-2 34225654 PMC8256493

[tpj70317-bib-0046] Nardmann, J. & Werr, W. (2012) The invention of WUS‐like stem cell‐promoting functions in plants predates leptosporangiate ferns. Plant Molecular Biology, 78(1–2), 123–134. Available from: 10.1007/s11103-011-9851-4 22076631

[tpj70317-bib-0047] Nietzel, T. , Elsässer, M. , Ruberti, C. , Steinbeck, J. , Ugalde, J.M. , Fuchs, P. et al. (2018) The fluorescent protein sensor roGFP2‐Orp1 monitorsin vivo H_2_O_2_ and thiol redox integration and elucidates intracellular H_2_O_2_ dynamics during elicitor‐induced oxidative burst in Arabidopsis. New Phytologist, 221(3), 1649–1664. Available from: 10.1111/nph.15550 30347449

[tpj70317-bib-0048] Noctor, G. , Cohen, M. , Trémulot, L. , Châtel‐Innocenti, G. , Van Breusegem, F. & Mhamdi, A. (2024) Glutathione: a key modulator of plant defence and metabolism through multiple mechanisms. Journal of Experimental Botany, 75(15), 4549–4572. Available from: 10.1093/jxb/erae194 38676714

[tpj70317-bib-0049] Noctor, G. , Reichheld, J.‐P. & Foyer, C.H. (2018) ROS‐related redox regulation and signaling in plants. Seminars in Cell & Developmental Biology, 80, 3–12. Available from: 10.1016/j.semcdb.2017.07.013 28733165

[tpj70317-bib-0050] Pak, V.V. , Ezeriņa, D. , Lyublinskaya, O.G. , Pedre, B. , Tyurin‐Kuzmin, P.A. , Mishina, N.M. et al. (2020) Ultrasensitive genetically encoded indicator for hydrogen peroxide identifies roles for the oxidant in cell migration and mitochondrial function. Cell Metabolism, 31(3), 642–653.e646. Available from: 10.1016/j.cmet.2020.02.003 32130885 PMC7088435

[tpj70317-bib-0051] Peláez‐Vico, M.A. , Fichman, Y. , Zandalinas, S.I. , Foyer, C.H. & Mittler, R. (2024) ROS are universal cell‐to‐cell stress signals. Current Opinion in Plant Biology, 79, 102540. Available from: 10.1016/j.pbi.2024.102540 38643747

[tpj70317-bib-0052] Queval, G. & Noctor, G. (2007) A plate reader method for the measurement of NAD, NADP, glutathione, and ascorbate in tissue extracts:: application to redox profiling during rosette development. Analytical Biochemistry, 363(1), 58–69. Available from: 10.1016/j.ab.2007.01.005 17288982

[tpj70317-bib-0053] Rahantaniaina, M.S. , Tuzet, A. , Mhamdi, A. & Noctor, G. (2013) Missing links in understanding redox signaling via thiol/disulfide modulation: how is glutathione oxidized in plants? Frontiers in Plant Science, 4, 477. Available from: 10.3389/fpls.2013.00477 24324478 PMC3838956

[tpj70317-bib-0054] Salbitani, G. , Bottone, C. & Carfagna, S. (2017) Determination of reduced and Total glutathione content in extremophilic microalga Galdieria phlegrea. Bio‐Protocol, 7(13), e2372. Available from: 10.21769/BioProtoc.2372 34541114 PMC8413652

[tpj70317-bib-0055] Salinitro, M. , Mattarello, G. , Guardigli, G. , Odajiu, M. & Tassoni, A. (2021) Induction of hormesis in plants by urban trace metal pollution. Scientific Reports, 11(1), 20329. Available from: 10.1038/s41598-021-99657-3 34645888 PMC8514553

[tpj70317-bib-0056] Schoof, H. , Lenhard, M. , Haecker, A. , Mayer, K.F.X. , Jürgens, G. & Laux, T. (2000) The stem cell population of Arabidopsis shoot meristems is maintained by a regulatory loop between the CLAVATA and WUSCHEL genes. Cell, 100(6), 635–644. Available from: 10.1016/s0092-8674(00)80700-x 10761929

[tpj70317-bib-0057] Schwarzländer, M. , Fricker, M.D. , Müller, C. , Marty, L. , Brach, T. , Novak, J. et al. (2008) Confocal imaging of glutathione redox potential in living plant cells. Journal of Microscopy, 231(2), 299–316. Available from: 10.1111/j.1365-2818.2008.02030.x 18778428

[tpj70317-bib-0058] Shanmugam, V. , Tsednee, M. & Yeh, K.‐C. (2012) ZINC TOLERANCE INDUCED BY IRON 1 reveals the importance of glutathione in the cross‐homeostasis between zinc and iron in Arabidopsis thaliana. The Plant Journal, 69(6), 1006–1017. Available from: 10.1111/j.1365-313X.2011.04850.x 22066515

[tpj70317-bib-0059] Shimamura, M. (2016) Marchantia polymorpha: taxonomy, phylogeny and morphology of a model system. Plant and Cell Physiology, 57(2), 230–256. Available from: 10.1093/pcp/pcv192 26657892

[tpj70317-bib-0060] Spencer, V. , Wallner, E.S. , Jandrasits, K. , Edelbacher, N. , Mosiolek, M. & Dolan, L. (2024) Three‐dimensional anatomy and dorsoventral asymmetry of the mature meristem develops from a symmetrical gemma meristem. Development (Cambridge, England), 151(23), dev204349. Available from: 10.1242/dev.204349 39545722 PMC11634034

[tpj70317-bib-0061] Sugano, S.S. , Nishihama, R. , Shirakawa, M. , Takagi, J. , Matsuda, Y. , Ishida, S. et al. (2018) Efficient CRISPR/Cas9‐based genome editing and its application to conditional genetic analysis in Marchantia polymorpha. PLoS One, 13(10), e0205117. Available from: 10.1371/journal.pone.0205117 30379827 PMC6209168

[tpj70317-bib-0062] Takahashi, G. , Betsuyaku, S. , Okuzumi, N. , Kiyosue, T. & Hirakawa, Y. (2021) An evolutionarily conserved coreceptor gene is essential for CLAVATA signaling in Marchantia polymorpha. Frontiers in Plant Science, 12, 657548. Available from: 10.3389/fpls.2021.657548 33927741 PMC8076897

[tpj70317-bib-0063] Tietze, F. (1969) Enzymic method for quantitative determination of nanogram amounts of Total and oxidized glutathione ‐ applications to mammalian blood and other tissues. Analytical Biochemistry, 27(3), 502–522. Available from: 10.1016/0003-2697(69)90064-5 4388022

[tpj70317-bib-0064] Tsukagoshi, H. , Busch, W. & Benfey, P.N. (2010) Transcriptional regulation of ROS controls transition from proliferation to differentiation in the root. Cell, 143(4), 606–616. Available from: 10.1016/j.cell.2010.10.020 21074051

[tpj70317-bib-0065] Ugalde, J.M. , Fuchs, P. , Nietzel, T. , Cutolo, E.A. , Homagk, M. , Vothknecht, U.C. et al. (2021) Chloroplast‐derived photo‐oxidative stress causes changes in H_2_O_2_ and EGSH in other subcellular compartments. Plant Physiology, 186(1), 125–141. Available from: 10.1093/plphys/kiaa095 33793922 PMC8154069

[tpj70317-bib-0066] Ugalde, J.M. , Schlößer, M. , Dongois, A. , Martinière, A. & Meyer, A.J. (2021) The latest HyPe(r) in plant H_2_O_2_ biosensing. Plant Physiology, 187(2), 480–484. Available from: 10.1093/plphys/kiab306 34608965 PMC8491017

[tpj70317-bib-0067] Vernoux, T. , Wilson, R.C. , Seeley, K.A. , Reichheld, J.‐P. , Muroy, S. , Brown, S. et al. (2000) The ROOT MERISTEMLESS1/CADMIUM SENSITIVE2 gene defines a glutathione‐dependent pathway involved in initiation and maintenance of cell division during postembryonic root development. The Plant Cell, 12(1), 97–109. Available from: 10.1105/tpc.12.1.97 10634910 PMC140217

[tpj70317-bib-0068] Wagner, S. , Steinbeck, J. , Fuchs, P. , Lichtenauer, S. , Elsässer, M. , Schippers, J.H.M. et al. (2019) Multiparametric real‐time sensing of cytosolic physiology links hypoxia responses to mitochondrial electron transport. New Phytologist, 224(4), 1668–1684. Available from: 10.1111/nph.16093 31386759

[tpj70317-bib-0069] Zeng, J. , Dong, Z. , Wu, H. , Tian, Z. & Zhao, Z. (2017) Redox regulation of plant stem cell fate. The EMBO Journal, 36(19), 2844–2855. Available from: 10.15252/embj.201695955 28838936 PMC5623875

[tpj70317-bib-0070] Zhou, H. , Huang, J.J. , Willems, P. , Van Breusegem, F. & Xie, Y.J. (2023) Cysteine thiol‐based post‐translational modification: what do we know about transcription factors? Trends in Plant Science, 28(4), 415–428. Available from: 10.1016/j.tplants.2022.11.007 36494303

[tpj70317-bib-0071] Zhou, L.‐Z. & Dresselhaus, T. (2023) CHAPTER Five ‐ Multiple roles of ROS in flowering plant reproduction. In: Mittler, R.O.N. & Breusegem, F.V. (Eds.) Advances in Botanical Research, Vol. 105. Amsterdam, Netherlands: Academic Press, pp. 139–176. Available from: 10.1016/bs.abr.2022.10.002

[tpj70317-bib-0072] Zhuravlev, A. , Ezerin, D. , Ivanova, J. , Guriev, N. , Pugovkina, N. , Shatrova, A. et al. (2024) HyPer as a tool to determine the reductive activity in cellular compartments. Redox Biology, 70, 103058. Available from: 10.1016/j.redox.2024.103058 PMC10848024 38310683

