# Supplementary material for: Redox buffering and H2O2 orchestrate the vegetative development of Marchantia polymorpha
Source: Plant J. 2025 Jul 18;123(2):e70317. doi: 10.1111/tpj.70317 (PMC12274077; doi:10.1111/tpj.70317)
Supplement: Supplementary file 1 — Figure S1. Subcellular localization of the biosensors. (a) Subcellular localization of roGFP2‐hGrx1 fluorescence. (b) Subcellular localization of HyPer7 fluorescence. Scale bars 10 μm. Figure S2. Visualization of the Mpgsh1 knockout gRNAs and quantification of the total GSH amount after BSO treatment. (a) Positions of the five gRNAs (red circle) in the MpGSH1 gene locus for the CRISPR‐Cas9 knockout approach. (b) The total GSH content of 28 DAG plants grown with or without 500 μM BSO. The GSH level was quantified using the DTNB recycling assay (n ≥ 5). Data are represented as mean ± standard deviation. Statistical significance was determined using the single ANOVA test followed by Tukey (P < 0.05). Figure S3. Detailed meristem analysis of the HyPer7 sensor in 7‐day‐old M. polymorpha plants. Movies visualize HyPer7 redox states in the meristematic region of M. polymorpha plants. (a) 3D reconstruction of a control plant, showing that a small zone with an oxidized HyPer7 state is localized in the center of an overall more reduced meristematic region. (b) 3D reconstruction of a plant treated with MpCLE2p, unveiling an increased number of expanded zones with higher HyPer7 oxidation formed in the extended and more reduced meristematic region. (c, d) Single‐plane movies for detailed observation of the oxidation dynamics within the meristematic region of control (c) and MpCLE2p‐treated (d) plants. [file TPJ-123-0-s001.pptx]

## Slide 1
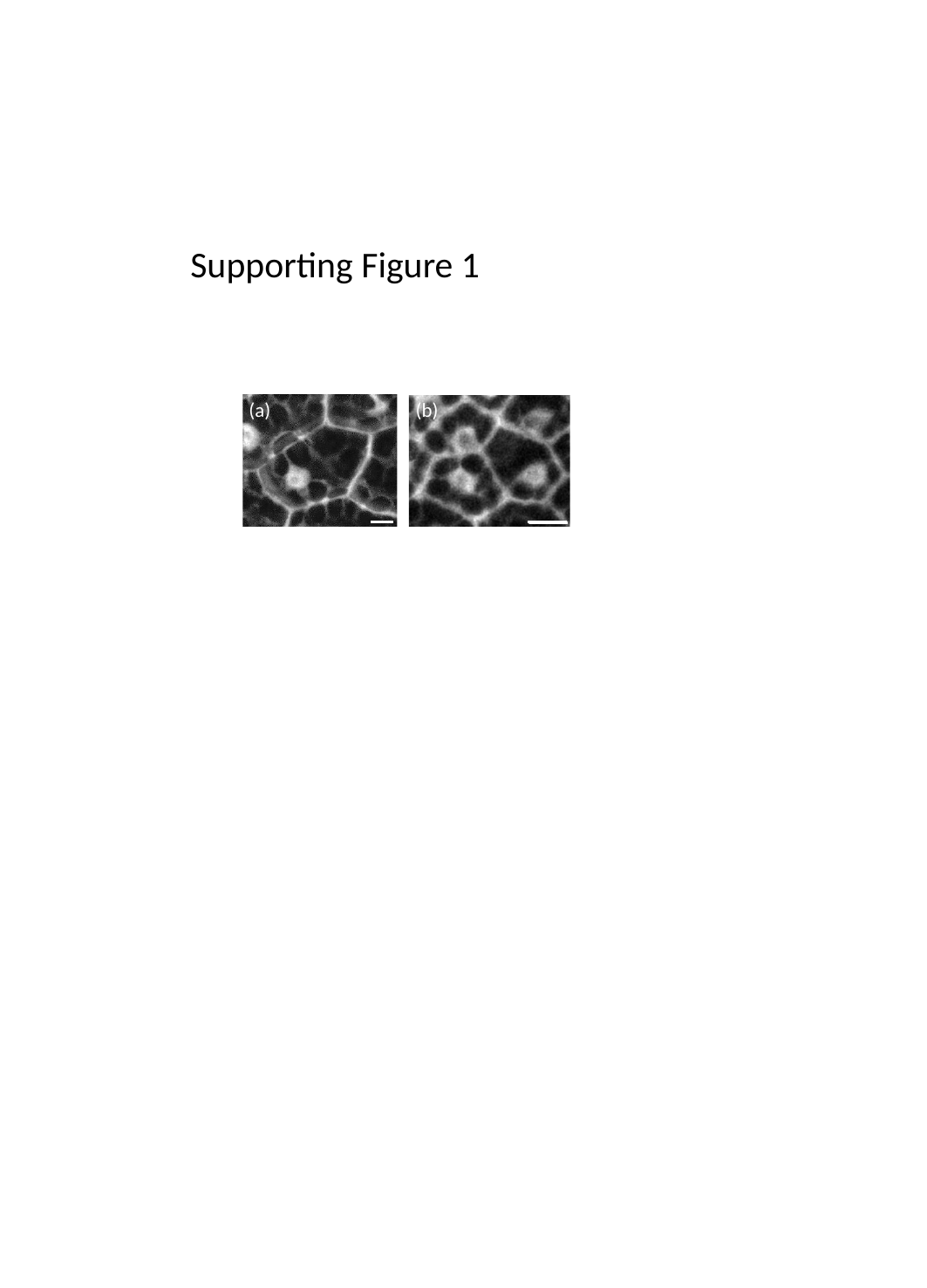

Supporting Figure 1
(a)
(b)

## Slide 2
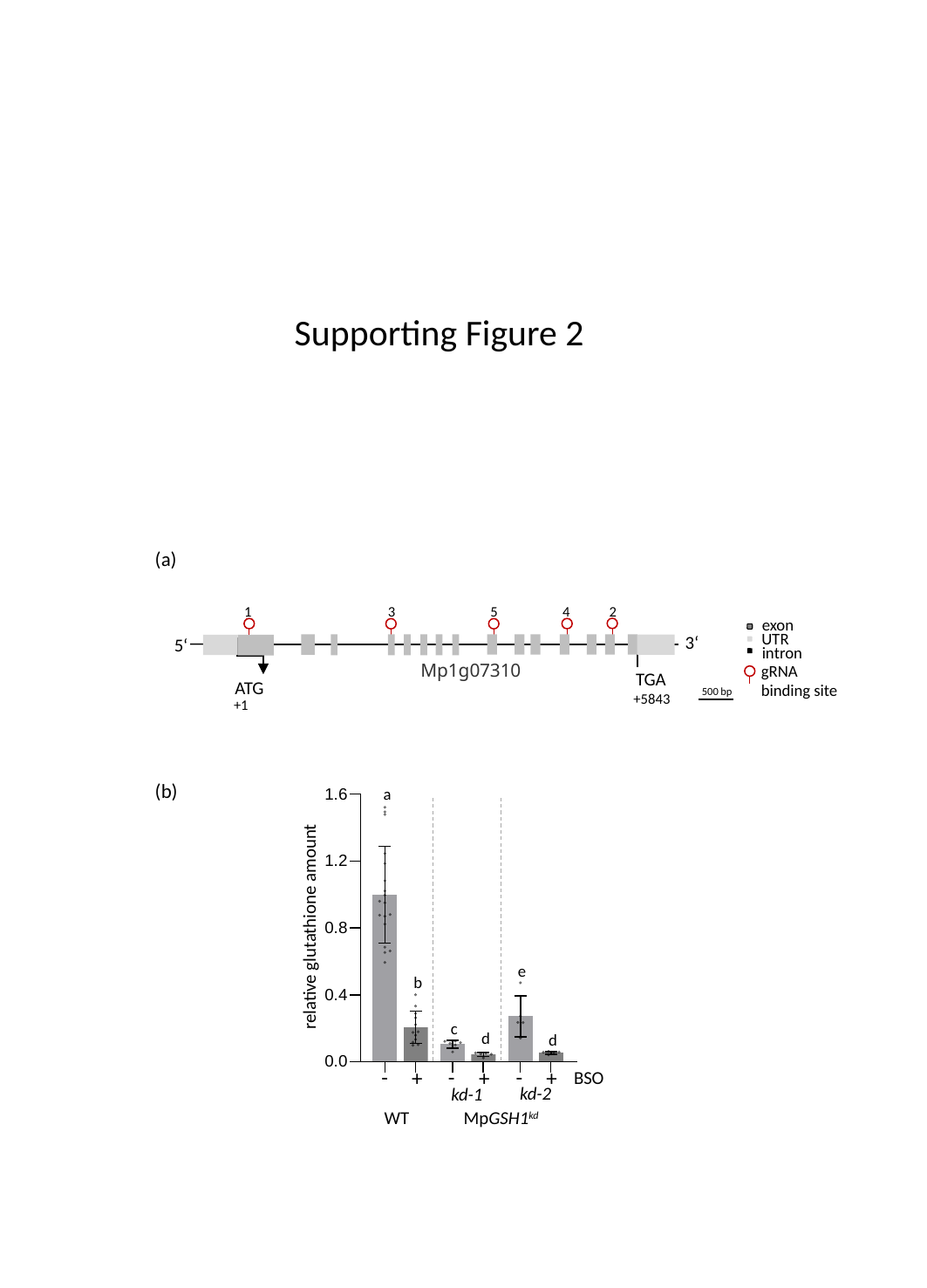

Supporting Figure 2
(a)
1
4
2
3
5
exon
UTR
intron
3‘
5‘
TGA
ATG
500 bp
+1
Mp1g07310
gRNA binding site
+5843
-
+
-
+
-
+
BSO
(b)
a
relative glutathione amount
e
b
c
d
d
kd-2
kd-1
WT
MpGSH1kd

## Slide 3
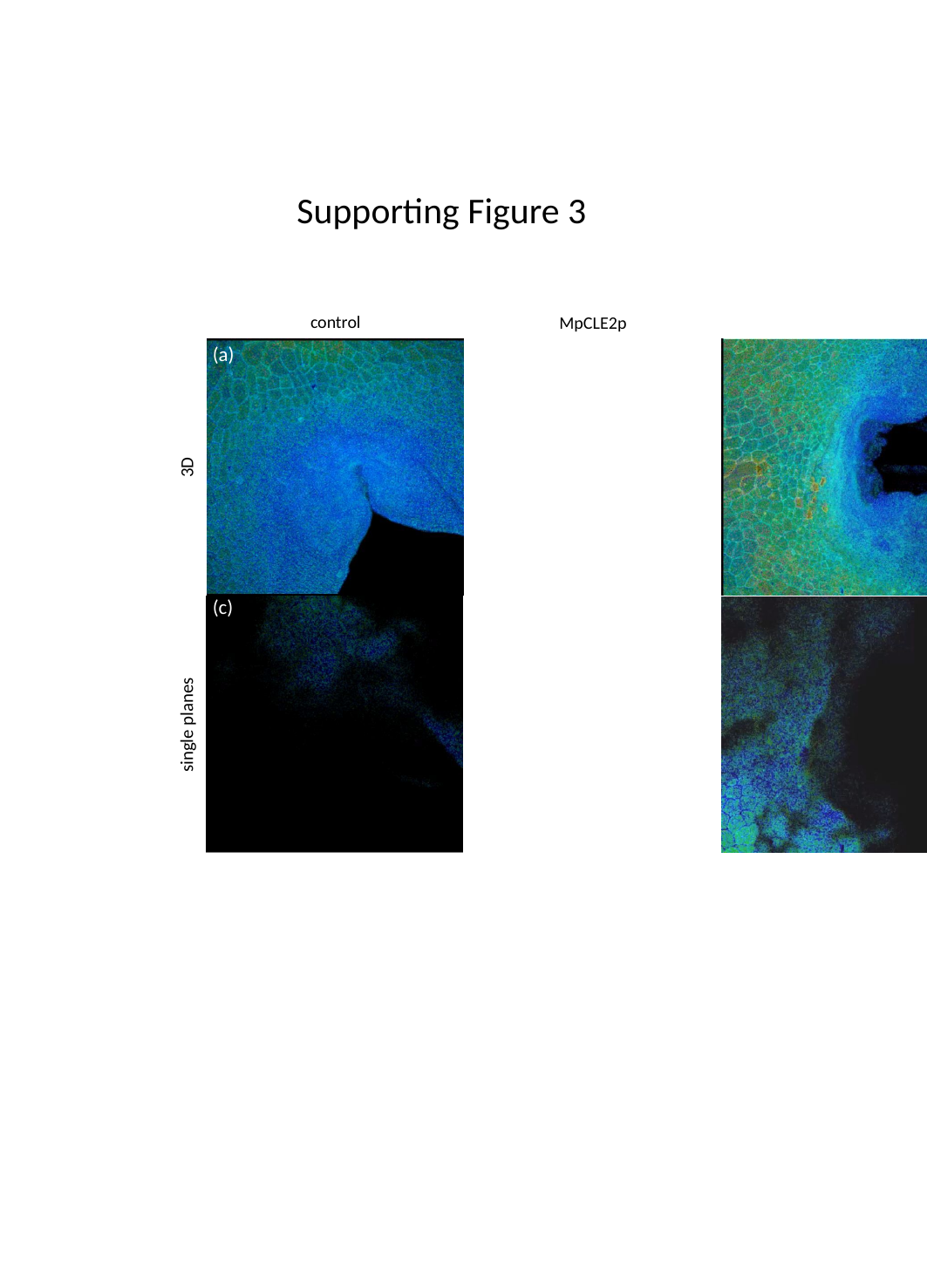

Supporting Figure 3
control
MpCLE2p
(a)
(b)
(b)
(a)
3D
(c)
(d)
(d)
(c)
single planes
