# Supplementary figures and images for: Redox buffering and H2O2 orchestrate the vegetative development of Marchantia polymorpha
Source: Plant J. 2025 Jul 18;123(2):e70317. doi: 10.1111/tpj.70317 (PMC12274077; doi:10.1111/tpj.70317)

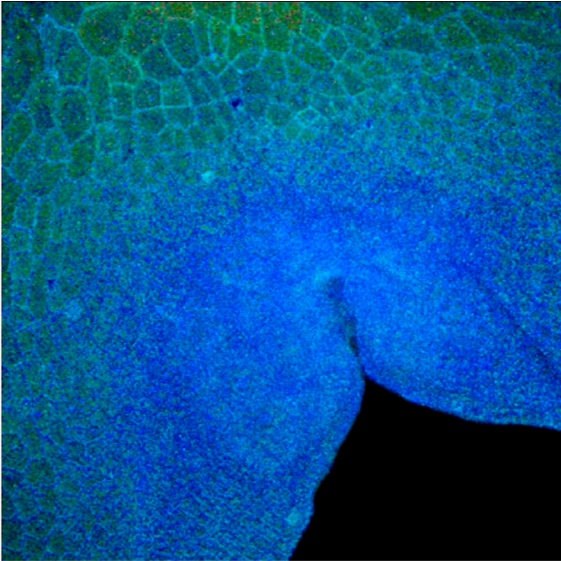

Supplement: Supplementary file 3 — Video S1. Video of Figure S3. [file TPJ-123-0-s002.zip › S3A.tif]

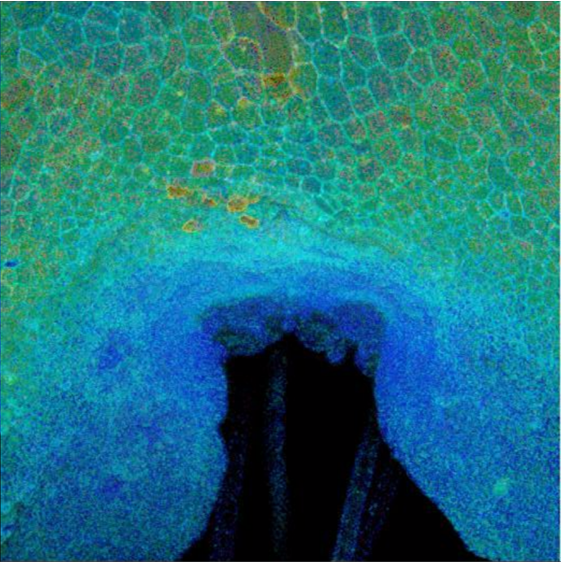

Supplement: Supplementary file 3 — Video S1. Video of Figure S3. [file TPJ-123-0-s002.zip › S3B.tif]

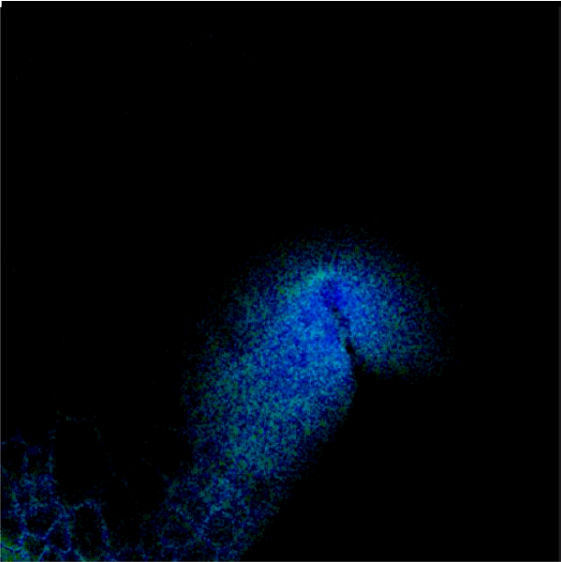

Supplement: Supplementary file 3 — Video S1. Video of Figure S3. [file TPJ-123-0-s002.zip › S3C.tif]

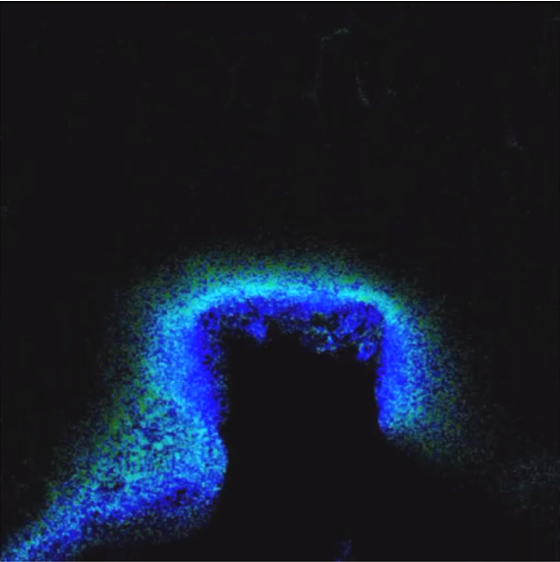

Supplement: Supplementary file 3 — Video S1. Video of Figure S3. [file TPJ-123-0-s002.zip › S3D.tif]
